# Design principles of ciliary signaling

**DOI:** 10.1242/jcs.264325

**Published:** 2025-10-24

**Authors:** Carolyn M. Ott, Jennifer Lippincott-Schwartz

**Affiliations:** Janelia Research Campus, Howard Hughes Medical Institute, Ashburn, VA 20147, USA

**Keywords:** Primary cilia, Bow-tie architecture, Network motifs, Robustness, Evolvability

## Abstract

Primary cilia are microtubule-based sensory organelles that have been conserved throughout eukaryotic evolution. As discussed in this Review, a cilium is an elongated and highly specialized structure, and, together with its ability to selectively traffic and concentrate proteins, lipids and second messengers, it creates a signaling environment distinct from the cell body. Ciliary signaling pathways adopt a bow-tie network architecture, in which diverse inputs converge on shared effectors and second messengers before diverging to multiple outputs. Unlike other cellular bow-tie systems, cells exploit ciliary geometry, compartmentalization and infrastructure to enhance sensitivity at multiple scales, from individual molecular reactions to entire signaling pathways. In cilia, integration of the bow-tie network architecture with their specialized structure and unique environment confers robustness and evolvability, which enables cilia to acquire diverse signaling roles. However, this versatility comes with vulnerability – rare mutations that disrupt the features most essential for cilia robustness cause multisystem ciliopathies.

## Teaser

**Summary:** A discussion of why the unique composition and geometry of cilia enables highly tunable, sensitive, robust and evolvable responses to environmental signals.

## Introduction

The last eukaryotic common ancestor (LECA) possessed flagella – tail-like organelles that enabled cell motility and environment sensing (Derderian et al., 2023; Satir et al., 2008). Primary cilia, which evolved from flagella, became specialized sensory organelles that have been evolutionarily conserved and are now widespread in mammals, typically appearing as a single cilium per cell (Sung and Leroux, 2013). Cilia are small elongated sub-compartments with a unique molecular composition positioned to sample the extracellular environment.

Across cell types and signaling pathways, cilia share a conserved architectural logic that enables them to integrate, process and transmit signals with remarkable specificity and adaptability. This organization underpins their role as cellular antennae – structures that orchestrate mammalian development, sustain tissue homeostasis, and coordinate neural and endocrine signaling via pathways initiated at the ciliary surface (Goetz and Anderson, 2010; Hilgendorf et al., 2024). Ciliary receptors sense a broad range of extracellular signals, including growth factors, lipids, neuromodulators, odors and even photons (Hilgendorf et al., 2019; Mykytyn and Askwith, 2017). These inputs are relayed by intermediate effectors within the cilium, driving outputs such as changes in gene expression, cell morphology, proliferation and membrane excitability (Anvarian et al., 2019). Well-studied examples include sonic hedgehog (SHH) signaling in limb patterning, cyst prevention by polycystins in the kidney, and sensory transduction in photoreceptors and olfactory neurons (Reiter and Leroux, 2017).

When cilia malfunction, the consequences are profound. Complete loss of cilia is embryonic lethal in mammals (Huangfu et al., 2003; Murcia et al., 2000; Nonaka et al., 1998) and impaired cilia function causes ciliopathies – multisystem disorders affecting many organs including kidney, liver, eye, lung and heart (Mill et al., 2023). In the brain, ciliary signaling defects contribute to obesity, cognitive impairment and neuropsychiatric disorders (Brewer et al., 2022; Guo et al., 2017; Khan et al., 2024; Kostyanovskaya et al., 2025 preprint).

Despite their established importance in health and disease, fundamental questions about cilia remain. Why is signaling through cilia distinct from signaling elsewhere in the cell, and why have cilia been retained through evolution and adapted for diverse, specialized sensory functions? In biology, questions can be divided into proximate (mechanistic ‘how’) and ultimate (evolutionary ‘why’) (Bergman and Beehner, 2022; Mayr, 1961). Much progress has come from proximate studies addressing how cilia assemble, emerge during development and transmit signals (Goetz and Anderson, 2010; Hilgendorf et al., 2024; Zhao et al., 2023). In this Review, however, we focus on the ultimate questions – the evolutionary and functional advantages of cilia.

To address these questions, we first describe the structure, geometry and composition of primary cilia. We then consider how these physical features interface with ciliary signaling network architecture (see Glossary). At every level, from individual reactions to regulatory modules and full pathways, the geometry, infrastructure, tunable composition and concentrating capacity of cilia enhance both sensitivity and robustness (see Glossary). Finally, we discuss the survival value of primary cilia and propose a new hypothesis for their evolutionary conservation across eukaryotes.

Glossary **Amplification:** a network motif that generates more output than input. Amplification can boost weak signals, overcome noise and effect the duration of pathway responses.

**Bow-tie architecture:** a network architecture in which diverse inputs converge on a conserved weakly linked processing module and then diverge to a variety of cellular outputs. This network architecture is robust and enables evolutionary innovation (Csete and Doyle, 2004; Kitano, 2004).

**Constraint and deconstraint:** constraints in evolution limit diversification and preserve core design elements. Deconstraint refers to the relaxation of these limitations, enabling diversification. However, to generate beneficial biological outcomes, deconstraint depends on underlying constraints, which anchor new variation to established, functional systems (Kirschner and Gerhart, 1998).

**Crosstalk:** interaction between signaling pathways in which one pathway influences the outcome of another, leading to either positive or negative modulation of signaling responses. Crosstalk mechanisms include cross activation (one pathway activates another), cross inhibition (one pathway suppresses another), synergistic effects (enhanced combined output), heterologous desensitization (see below) (loss of response due to the activity of another pathway) and effector competition (see below) (Vert and Chory, 2011; Wang and Ogden, 2025).

**Effector competition:** effectors are proteins downstream of the receptor in a signaling pathway (such as G proteins, kinases or phosphatases). Effector competition is a specific type of crosstalk in which the signal output of one pathway is limited because another pathway monopolizes or sequesters shared downstream effectors (Brinkerhoff et al., 2008).

**Evolvability:** the capacity of a biological system to generate and select for novel functional traits under evolutionary pressures. Highly evolvable systems minimize the potential for lethal outcomes and require fewer changes to generate diversity. Redundancy, versatile protein elements, weak linkage, exploratory mechanisms and compartmentalization contribute to evolvability (Kirschner and Gerhart, 1998).

**Feedforward:** a network motif in which a signaling pathway branches and both branches converge on a downstream output – either independently or in coordination. One branch can drive the output while the other modulates its timing or strength, creating sustained activity (Alon, 2007; Goentoro et al., 2009; Mangan and Alon, 2003). Feedforward motifs can be coherent, where both branches exert the same effect on the downstream target (e.g. both activate or both inhibit), leading to sustained or reinforced signaling; or incoherent, where branches exert opposing effects (one activates, one inhibits), which can sharpen response timing, create pulse-like outputs, or buffer against noise. G proteins often participate in such loops – Gα activates one branch while Gβγ activates another (Logothetis et al., 1987); however, the signaling configuration only qualifies as a feedforward motif if both branches converge on a single output (Alon, 2007).

**Fragile point:** a specific feature or component within a robust biological system that becomes a critical vulnerability under unexpected or rare perturbations. Although robust systems are designed to withstand common disturbances, their optimized structure can lead to single points of failure – the ‘fragile points’ – that, when compromised, result in dramatic system malfunction or collapse (Csete and Doyle, 2004; Kitano, 2004).

**Heterologous desensitization:** a form of signaling crosstalk in which activation of one receptor pathway promotes the internalization or downregulation of different, unrelated receptors, resulting in simultaneous decreases in responsiveness to multiple signals (von Zastrow, 2003).

**Kinetic compartmentalization:** a mechanism in which key signaling pathway components – such as receptors, enzymes, or channels – are present at very high local concentrations relative to their diffusible second messengers inside a subcellular microdomain, such as a cilium or flagellum (Trötschel et al., 2020).

**Multi-step ultrasensitivity:** a phenomenon in which the cumulative arrangement of several signaling steps creates a highly sigmoidal, switch-like response to stimuli at the pathway level. Unlike classical allostery or cooperative binding, multi-step ultrasensitivity arises when sequential reactions depend strictly on the product of the preceding step, producing a sharp transition from off to on as input increases (Ferrell and Ha, 2014b; Trötschel et al., 2020).

**Negative feedback:** a network motif in which the output of a signaling pathway acts to suppress or dampen earlier steps in the same pathway. Negative feedback reduces fluctuations, stabilizes pathway activity, and enables robust adaptation, ultimately shaping both the timing and magnitude of cellular responses (Brandman and Meyer, 2008).

**Network architecture:** the overarching organizational principles that define how components of signal transduction pathways are connected, interact and process information. Network architecture encompasses many signaling pathways and the organization of regulatory motifs (Barabási and Oltvai, 2004).

**Noise:** any variation or fluctuation in the activity of a signaling pathway that is not directly caused by genuine, intended input signals. Noise arises from the inherent stochasticity of biochemical reactions (intrinsic noise) as well as from unrelated cellular processes or environmental fluctuations (extrinsic noise). In biological networks, noise can obscure weak signals or cause erroneous activation and can be filtered or managed by network motifs such as feedback and compartmentalization to preserve signaling fidelity and information transmission (Ladbury and Arold, 2012; Shibata and Fujimoto, 2005).

**Positive feedback:** a network element in which the output of a signaling pathway stimulates processes that enhance or reinforce the activity of upstream events in the same pathway causing signal amplification (Brandman and Meyer, 2008; Ferrell, 2002; Xiong and Ferrell, 2003).

**Receptor reserve:** a signaling system configuration in which maximal pathway output can be achieved even when a significant fraction of receptors remains unbound or inactive. This occurs when receptors are present in excess of the available downstream effectors (Hoyer and Boddeke, 1993; Zhu, 1993).

**Robustness:** the capacity of a biological system to sustain structural integrity or stable signaling outputs despite mutations, fluctuations, noise, or perturbations in its environment. Redundancy, modularity, decoupling, weak linkages and system control contribute to robustness (Blüthgen and Legewie, 2013; Kitano, 2004).

**Sensitivity:** the precision and efficiency with which a signaling pathway or molecular reaction generates a change in output upon a change in input (Ferrell and Ha, 2014a).

**Substrate channeling:** a mechanism by which the product of one enzymatic reaction is directly transferred to the next enzyme in a metabolic or signaling pathway, minimizing diffusion into the bulk environment. Substrate channeling ensures rapid, localized, and efficient signal propagation, reducing noise and loss, increasing pathway fidelity, and enabling high signal amplification without the need for globally elevated metabolite concentrations (Spivey and Ovádi, 1999).

**Ultrasensitivity:** a property of signaling systems where the output remains minimal at low input levels but rises sharply to a maximum once the input exceeds a defined threshold. This produces a highly sigmoidal dose-response relationship, resembling responses seen in cooperative ligand binding. Ultrasensitivity enables pathways to switch on and off quickly as input moves above or below the threshold (Ferrell and Ha, 2014a,b; Ferrell et al., 2014).

**Weak linkages:** a pathway design in which connections between reactions or pathway nodes are characterized by minimal mutual dependence or coupling (Kirschner and Gerhart, 1998; Kitano, 2004).

**Zero-order reaction:** an enzymatic reaction occurring at substrate-saturating concentrations, where the active sites of the enzyme are fully occupied and the reaction rate remains constant, independent of additional substrate. This contrasts with first-order and second-order (Michaelis–Menten) kinetics, where reaction rate increases linearly with substrate concentration at non-saturating substrate levels (Ferrell and Ha, 2014a; Goldbeter and Koshland, 1981).

**Zero-order ultrasensitivity:** a pathway response generated when two opposing enzymes operate under saturated (zero-order) conditions, producing a steep, switch-like (sigmoidal) change in substrate modification in response to small input changes. This mechanism can yield Hill coefficients greater than 2, reflecting sharp, cooperative-like transitions, but results from enzyme saturation and pathway design and not direct molecular cooperativity (Ferrell and Ha, 2014a; Goldbeter and Koshland, 1981).

## Ciliary physical architecture and composition

### Cilia ultrastructure

**Fig. 1.**
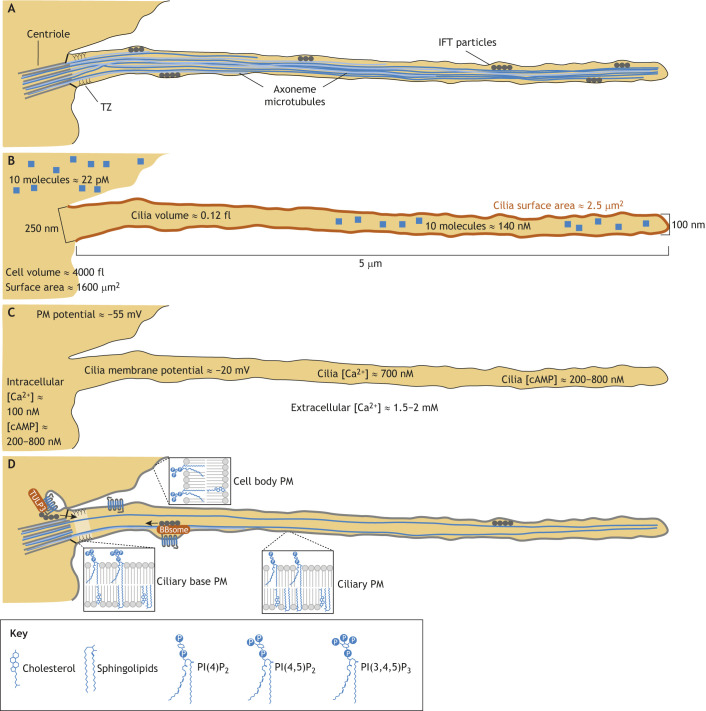
**Cilia physical architecture and composition.** (A) Ciliary axoneme MTs extend directly from the centriole. In primary cilia, MT doublets transition into singlets, with some terminating before reaching the ciliary tip. Motor proteins associate with IFT particles to facilitate cargo movement along the axoneme. At the cilium base, the TZ contains repeating Y-shaped protein complexes that establish and maintain the selective barrier, isolating the ciliary composition from the rest of the cell. (B) Cilia are small compared to the cell body and taper from base to tip. In this model, a cilium is 5 μm long, with a diameter of 250 nm over the first 1.5 μm, tapering to 100 nm across the next 1 μm, and maintaining a 100 nm diameter for the remaining 2.5 μm. These measurements also inform estimates of the molecular concentrations of 10 representative molecules within the cilium. (C) The ciliary ion concentration can differ markedly from that of the cell body, as demonstrated by measurements in cultured cells (Delling et al., 2013). These differences contribute to the unique ciliary signaling environment. Other small molecules, such as cAMP, have similar baseline concentrations in the cilium to that in cell body but independently change concentration in response to pathway activation. (D) The ciliary membrane is enriched in cholesterol, sphingolipids and the phosphoinositide PI(4)P, whereas PI(3,4,5)P_3_ is specifically concentrated in the TZ. In contrast, PI(4,5)P_2_ is present mainly in the PM of the rest of the cell and at the cilium base. The distinct ciliary protein composition is maintained through directed transport mechanisms, which involve TULP3, IFT and the BBSome.

Ciliary structure is central to the distinctiveness of ciliary signaling. Cilia are slender, membrane-encapsulated microtubule (MT)-based structures, which extend from a plasma membrane (PM)-anchored centriole. Ciliary MTs are also referred to as the axoneme. Unlike other organelles, the ciliary membrane and cilioplasm (ciliary cytoplasm) are continuous with the PM and cytoplasm (Fig. 1A). At the base, the transition zone (TZ) forms a selective gateway for protein traffic, although small proteins and other molecules can pass unrestricted (Garcia-Gonzalo and Reiter, 2017). Here, Y-shaped protein complexes form a bridge between the membrane and the axoneme (Park and Leroux, 2022). TZ proteins, such as nephronophthisis (NPHP) and Meckel–Gruber syndrome (MKS) proteins, localize to this boundary and help isolate the cilioplasm (Reiter and Leroux, 2017). Septins and a condensed lipid environment reinforce this boundary (Hu et al., 2010; Vieira et al., 2006).

Axonemal MTs begin as doublets at the cilium base, with plus ends oriented toward the tip. Kinesin- and dynein-driven intraflagellar transport (IFT) complexes create a regulated transport system (Pigino, 2021). Selective retention at the base and active transport to the tip contribute to distinct microenvironments within cilia (Hansen et al., 2025).

### Cilia size and shape

Cilia size and shape strongly influence signaling by determining surface area and volume (see Fig. 1B for representative dimensions) (Kiesel et al., 2020; Ott et al., 2024b). Although cilia across cell types share basic features, length varies widely – from less than 1 μm to over 20 μm (Ott et al., 2024a,b). Within a given cell type, cilia are usually similar in length, although setpoint mechanisms are not fully understood. Notably, signaling pathway activation can alter cilia length (Besschetnova et al., 2010).

Primary cilia are widest at the base (∼250 nm), tapering down to 100 nm or smaller (Kiesel et al., 2020; Ott et al., 2024b) as MTs transition from doublets to singlets or terminate (Kiesel et al., 2020; Ott et al., 2024b; Wang et al., 2025). Tapering varies between and within cell populations. Some cilia have a small membrane bulb at the tip, which is possibly involved in specialized signaling (Menco and Farbman, 1987; Müller et al., 2024).

Cilia are extremely small compared to cells. A 5 µm cilium has an estimated volume of ∼0.12 fl (see Fig. 1B), whereas a typical cultured cell is ∼4000 fl – more than 30,000 times larger (Puck et al., 1956). The surface area of a cilium (estimated at 2.5 µm^2^), is ∼650 times smaller than that of the cell (1600 µm^2^) (Girshovitz and Shaked, 2012). Subcompartments, like the ciliary tip, are even smaller. Importantly, cilia have a surface area-to-volume ratio ∼50 times larger than the rest of the cell. This geometry might exploit the advantages of dimensional reduction (Adam and Delbrück, 1968), whereby constraining molecular interactions to two-dimensional membrane surfaces can accelerate signaling. Although this principle applies to any membrane-localized signaling, cilia might amplify this advantage through extreme surface area-to-volume ratios and selective protein concentration, increasing receptor-effector interaction frequency.

### Concetrations of small molecules

Because of the small volume of the cilium, high ciliary concentrations can be reached more rapidly than in the cell body (Fig. 1B). A single molecule in a 0.12 fl cilium corresponds to ∼14 nM, whereas the same concentration in a 4000 fl cell requires >30,000 molecules. Fig. 1C compares ciliary and cytoplasmic Ca^2+^, cAMP and membrane potential. Resting Ca^2+^ concentration is ∼7-fold higher in cilioplasm than in cytoplasm (Delling et al., 2013). Small molecules like Ca^2+^ diffuse readily down their concentration gradients, so the elevated concentrations within cilia must be continually sustained by ciliary transporters (DeCaen et al., 2013; Delling et al., 2013; Jiang et al., 2019). Only when cytoplasmic Ca^2+^ exceeds ciliary concentrations will the diffusion gradient reverse; thus, the ciliary ionic distinctness is likely maintained under most physiological conditions.

Ca^2+^ levels can modulate the activity of adenylyl cyclases (ACs), which convert ATP into cyclic adenosine monophosphate (cAMP). cAMP is a key second messenger, activating ciliary protein kinase A (PKA). In olfaction, the isoform AC3 is central to signaling (Wong et al., 2000), although other AC isoforms also function in primary cilia (Bishop et al., 2007; Vuolo et al., 2015). Notably, Ca^2+^ promotes cAMP production by AC3 but inhibits AC5 and AC6 (Halls and Cooper, 2011). A-kinase anchoring proteins (AKAPs) scaffold PKA near ACs, forming multi-protein complexes that facilitate localized signaling (Choi et al., 2011; Kultgen et al., 2002).

Like Ca^2+^, cAMP can diffuse into and out of the cilium base. Initial studies reported >4 μM ciliary cAMP (Moore et al., 2016); however, subsequent experiments using improved strategies found ciliary cAMP levels were 200–800 nM – similar to those in the cytoplasm (Jiang et al., 2019). Although advantageous for signaling, the small ciliary volume can make measurements using fluorescent sensors challenging (Kierzek et al., 2021) (in the cilium, 200–800 nM would be the equivalent of 15–60 molecules). cAMP levels can increase in response to ciliary receptor activation (Jiang et al., 2019) and changes in ciliary cAMP are closely linked to downstream signaling and pathway activation (Hilgendorf et al., 2019; Paolocci and Zaccolo, 2023).

Ciliary membrane potential can differ from that of the rest of the cell. In cultured retinal pigment epithelial cells, the ciliary membrane potential is ∼30 mV less negative than that of the cell body (Delling et al., 2013). Because membrane potential varies significantly by cell type and depends on the specific ionic environment and channel complement (Binggeli and Weinstein, 1986), more measurements are needed to determine whether the magnitude of this membrane potential difference varies across cell types. Many channels localize to cilia, including nonselective ion channels, such as PC2 and transient receptor potential (TRP) channels (Pablo et al., 2017), which facilitate Na^+^/K^+^/Ca^2+^ flux, thus contributing to resting membrane potential.

### Cilia protein and lipid composition

Cilia exclude ribosomes and organelles, such as the endoplasmic reticulum (ER), Golgi, lysosomes, peroxisomes and mitochondria, so ciliary proteins must be synthesized elsewhere and transported in. The selective filter at the cilium base restricts both protein entry and exit, creating a distinct molecular composition that varies across tissues and cell types to support their diverse functions.

Several mechanisms regulate ciliary composition (Fig. 1D). Membrane proteins arrive via vesicle fusion or partitioning from the PM and enter by associating with the adaptor protein TUB like protein 3 (TULP3) and IFT (Hilgendorf et al., 2024). Soluble proteins utilize nuclear import proteins and IFT (Johnson and Malicki, 2019). IFT has an important role moving proteins within the cilium (Lacey and Pigino, 2025). The BBSome, which is a complex of eight Bardet–Biedl syndrome (BBS) proteins, binds to IFT proteins to facilitate activated membrane protein exit (Yang et al., 2020).

Lipid composition is likewise specialized. The inositol polyphosphate-5-phosphatase (INPP5E) converts phosphatidylinositol-4,5-bisphosphate [PI(4,5)P_2_] into phosphatidylinositol 4-phosphate (PI(4)P) at the cilium base. This is important for both targeting and activation because PI(4,5)P_2_ triggers release of certain proteins from carriers and is required for the proper function of many ion channels and transporters. In addition to PI(4,5)P_2_, phosphatidylinositol (3,4,5)-trisphosphate (PI(3,4,5)P3) is also enriched at the TZ (Conduit et al., 2021). The ciliary membrane is also rich in cholesterol and sphingomyelin (Nechipurenko, 2020). The unique ciliary membrane lipid composition modulates channel activity (Nechipurenko, 2020) and can change in response to signaling or intracellular changes.

Ciliary geometry and molecular composition are not just permissive for signaling; they are essential to how cilia function. As we address the ‘why’ questions, it becomes clear that cilia size, shape, structure and composition underpin every explanation.

## Bow-tie network architecture in cilia

**Fig. 2.**
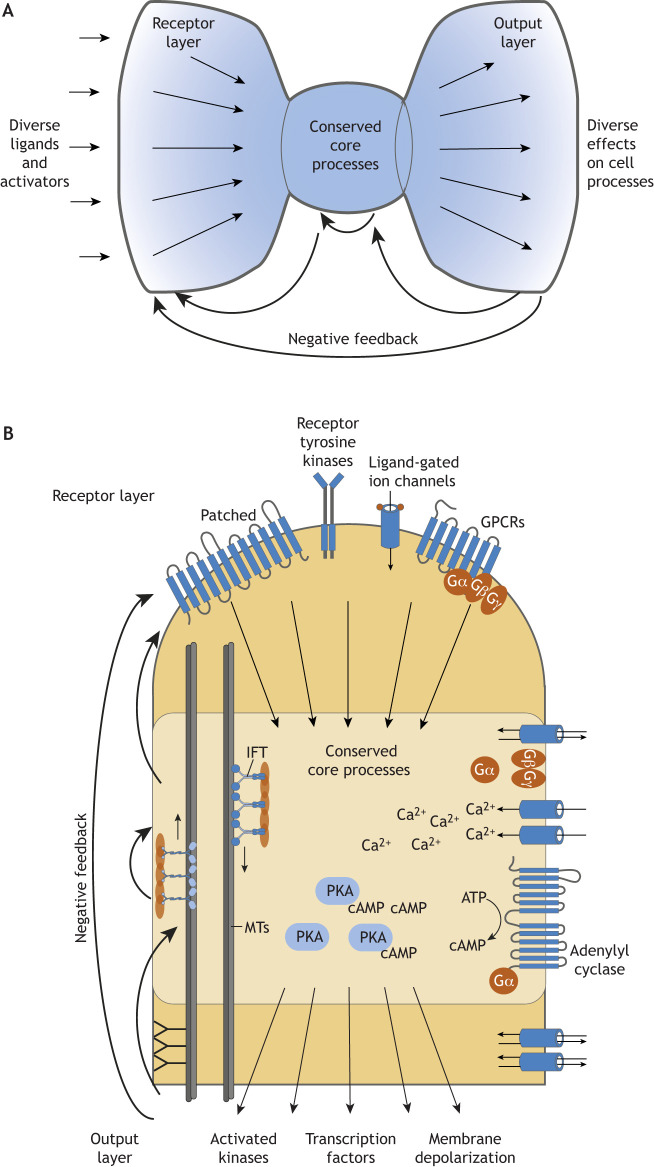
**Bow-tie network architecture of cilia signaling.** (A) Schematic of a bow-tie network architecture, in which diverse inputs converge on a shared set of effector proteins and second messengers – the bow-tie ‘knot’ – before diverging to multiple downstream outputs. Feedback loops (curved arrows) are integral to the organization and regulation of this network. Feedforward loops (not shown) contribute to the general signal flow (left-to-right arrows). (B) The ciliary bow-tie network architecture is embedded within the physical structure of the cilium. The receptor layer, which includes receptors such as PTCH1, RTKs, ligand-gated ion channels and GPCRs, activates secondary messengers like G proteins, which in turn activate downstream effectors. Here, we show G_βγ_ promoting opening of ion channels and G_α_ activation of AC3. These, along with subsequent effectors like cAMP and PKA, operate within the cilium. Once modified, terminal effectors leave the cilium. Ion channels are depicted in the output layer because they generate membrane depolarization in sensory cilia. Specialized ciliary features, including IFT, are integrated as regulatory elements within this network architecture.

Ciliary signaling pathways are typically organized into three functional layers: (1) the receptor layer, where diverse receptors on the ciliary surface detect extracellular ligands; (2) the processing layer, where downstream effectors operate within the cilium using a shared molecular machinery; and (3) the output layer, where terminal effectors trigger diverse cellular responses. This three-layer network architecture is common in signaling pathways. When diverse inputs funnel into a conserved, streamlined core before diverging into an array of outputs, the organization is called ‘bow-tie’ architecture (see Glossary; Fig. 2A) (Csete and Doyle, 2004; Kitano, 2004). At the bow-tie ‘knot’, input signals are compressed into a shared molecular machinery. Feedback and other network design elements (see below) operate within and between layers to ensure sensitive, robust responses.

Although bow-tie network architectures exist elsewhere in the cell, we propose that compartmentalization of the receptor and processing layers in cilia enables pathways within this network architecture to incorporate cilia-specific regulatory elements and leverage the small volume, high surface-area-to-volume ratio and distinct ciliary ion, protein and lipid environment. Fig. 2B illustrates the bow-tie architecture in ciliary signaling. Receptors, such as receptor tyrosine kinases (RTKs), ligand-gated channels and G-protein coupled receptors (GPCRs), initiate signal transduction. These receptors utilize common effectors, including second messengers, G proteins, PKA and ion channels. For the pathways characterized to date, terminal effectors (such as transcription factors or kinases modified by upstream effectors) leave the cilium (Anvarian et al., 2019); in the case of vision and olfaction, signaling causes membrane depolarization that travels to the cell body. Notably, of all known pathways with bow-tie architecture, only ciliary pathways incorporate IFT- and BBSome-mediated transport.

As we will explore in the following sections, the reason ciliary signaling differs from other cellular signaling pathways is that ciliary network architecture incorporates the advantages of ciliary physical architecture and composition.

## Cilia composition tunability

Unlike other organelles, the cilium controls molecular access through a single highly regulated gateway that selectively filters and transports components. The same features that restrict entry also function as a retention filter, maintaining high signaling molecule concentrations (Ye et al., 2018). Mutations disrupting TZ structure or filtering cause ciliopathies, underscoring the central role of this gateway (Reiter and Leroux, 2017).

The specialized architecture and trafficking systems that control which molecules enter, remain or exit distinguish ciliary tuning from that of the rest of the cell. Protein localization in the receptor layer, as well as downstream effector membrane proteins, follows the general biosynthetic pathway of other surface proteins but requires additional steps for ciliary targeting and passage through the TZ. These mechanisms utilize the IFT machinery and the unique ciliary lipid composition. Tubby family protein adaptors, including TULP3, bind cargo in PI(4,5)P_2_-rich membranes at the base, linking them to IFT subcomplexes that ferry them through the TZ (Mukhopadhyay et al., 2010). Inside the cilium, the PI(4)P-high, PI(4,5)P_2_-low environment triggers adaptors to release cargo (Garcia-Gonzalo et al., 2015).

Membrane protein export also utilizes IFT and the BBSome complex. Upon ligand binding, receptors are phosphorylated and ubiquitylated by E3 ligases. The BBSome is important for receptor recognition and removal (Ye et al., 2018) and mutations in BBS proteins cause GPCR accumulation and aberrant signaling (Reiter and Leroux, 2017). A recent study identified cilia and flagella associated protein 36 (CFAP36) as an IFT adaptor that carries ubiquitylated receptors only on retrograde IFT particles (Lange et al., 2025). Distinct from many signaling pathways, ubiquitylated activated receptor internalization in cilia is regulated by dynein-mediated IFT.

The bow-tie architecture makes signaling pathway specialization possible. For example, rod and cone photoreceptor cilia have evolved exceptionally complex signaling and high-volume trafficking, enabling precise localization and rapid phototransduction machinery turnover (Pearring et al., 2013). In addition, individual signaling pathways use specific adaptor proteins – melanocortin receptor accessory protein (MRAP2) enhances ciliary localization of the appetite-regulating GPCR, melanocortin 4 receptor (MC4R) (Bernard et al., 2023); GPR45 transports Gα_s_ into hypothalamic cilia to effect food intake regulation (Xun et al., 2025); and SHH signaling is tuned by shifting the balance of ciliary versus non-ciliary Patched 1 (PTCH1), Smoothened (SMO) and GPR161 pools, altering transcriptional repression thresholds (Hilgendorf et al., 2024; Hwang et al., 2021).

An advantage of the small size and efficient targeting and retrieval mechanisms in the cilium is that its composition can be rapidly reshaped. There is no universal ciliary proteome; rather, ciliary composition can differ both within and across cell populations (Hansen et al., 2025). This feature is utilized in network design elements in ciliary signaling. Importantly, ciliary composition tuning begins during biogenesis – the TZ and IFT not only build cilia but also determine which proteins enter and are retained. Their dual roles in assembly and ongoing tuning enable structural integrity, functional versatility and exceptional signaling sensitivity.

## Sensitivity in ciliary signaling

Ciliary signaling can be amazingly sensitive – photoreceptors can detect single photons and sea urchin sperm flagella can respond to single chemoattractant molecules. How is such sensitivity achieved? Pathway sensitivity is the culmination not only of each reaction in the pathway, but also how those reactions are organized and regulated. For many ciliary signaling pathways, we have an incomplete understanding of pathway regulation and precision. In this section, we discuss different ways that ciliary size, shape, structure and regulated composition can modulate individual reaction sensitivity, network elements and ultimately whole pathways.

### Individual reaction sensitivity

Signal transduction relies fundamentally on binding and dissociation events between interacting molecules, including receptor–ligand, protein–ion, protein–lipid and protein–protein interactions. The concentrating power of cilia can increase the probability of inter-molecular binding. This is exemplified in sea urchin sperm flagella and photoreceptors, which harbor 400,000 and 60 million receptors, respectively (Skiba et al., 2023; Trötschel et al., 2020) – densities optimized for maximal ligand or photon capture.

Selective membrane protein localization can create differences between ciliary and non-ciliary signaling – when ciliary receptors have higher ligand affinity than their non-ciliary counterparts, ciliary detection is more sensitive. The neuropeptide Y (NPY) receptor Y2 (NPY2R) binds its ligand ∼30-fold more tightly than the NPY receptor Y1 (NPY1R) [dissociation constant (*K*_D_)=0.12 nM versus 4.0 nM, respectively] (Tang et al., 2022). Because only NPY2R localizes to cilia (Loktev and Jackson, 2013), at low concentrations, ciliary NPY detection is more sensitive than detection at the cell surface. For each signaling pathway, sensitivity differences between cilia and cell surface isoforms depend on receptor *K*_D_.

Concentrating second messengers and effector proteins in cilia can change enzyme reaction kinetics. When substrate is not saturating, the reaction rate changes as substrate concentration increases. However, high substrate concentrations – like those possible in cilia – shift enzyme reactions to zero-order kinetics (see Glossary) where the reaction proceeds at the maximal rate and product formation is insensitive to fluctuations in substrate concentration (Ferrell and Ha, 2014a). A single zero-order reaction is less sensitive to substrate fluctuations; however, combining zero-order reactions in a signaling pathway can increase overall pathway sensitivity, as detailed below (Ferrell and Ha, 2014a).

The small, crowded environment in cilia promotes formation of complexes among pathway components, enabling substrate channeling (see Glossary). In addition to enhancing signal fidelity and minimizing diffusion delays (Sweetlove and Fernie, 2018; Tullman-Ercek, 2015), enzyme coupling can induce conformational changes that increase reaction rates and efficiency (Tulsian et al., 2017). In sea urchin sperm flagella, cGMP is transferred from cyclic nucleotide-gated channels directly to phosphodiesterases at rates faster than those generated by free diffusion (Pichlo et al., 2014; Trötschel et al., 2020).

Such direct hand-off is distinct from kinetic compartmentalization (see Glossary), wherein the unique geometry and high ciliary signaling protein concentrations (compared to freely diffusing messengers) permit highly efficient signaling even without direct enzyme–enzyme contact. In sperm chemosensing, receptors and effectors are present at micromolar concentrations, whereas freely diffusing second messengers like cGMP, cAMP, H^+^ and Ca^2+^ are up to 1000-fold less abundant (Trötschel et al., 2020). Although both substrate channeling and kinetic compartmentalization can occur elsewhere in the cell, the specialized composition and architecture of cilia make them especially conducive to these sensitivity- and fidelity-increasing configurations.

Reaction sensitivity and duration within cilia are influenced by more than just receptor affinity or enzyme kinetics. Ion-binding proteins can prolong signaling by slowing diffusion and reduce the magnitude of concentration changes during ion influx (Eisner et al., 2023). In thin elongated cilia, the membrane can be densely packed with ion-binding proteins and the high surface area-to-volume ratio allows for extremely high buffering capacity (Eisner et al., 2023). For example, uncaged Ca^2+^ and cAMP in olfactory neuronal cilia persist locally instead of rapidly diffusing away (Takeuchi and Kurahashi, 2018). Spatially confined and temporally sustained signals can enhance sensitivity by creating opportunities for amplification, a topic discussed in the next section.

### Network design elements

Cellular responses depend not only on individual reaction kinetics but also on how reactions are organized into signaling pathways. The arrangement and interconnection of reactions – referred to as network elements or motifs – determines how signals are processed, integrated and relayed within cells. Network elements influence how signals from different parts of a pathway interact and contribute to the overall output. Below, we highlight key network elements in ciliary signaling – amplification, negative feedback and feedforward loops, and crosstalk – and discuss how the cilium uniquely enhances their specificity and efficiency, thereby providing a foundation for understanding pathway-level sensitivity.

### Amplification

Each of the three cilia signaling cascades that have been quantitatively elucidated utilizes the advantages of cilia to amplify signals. In olfactory sensory cilia, weak odorant stimuli can trigger a two-stage amplification cascade. First, odorant binding to GPCRs activates G proteins, leading to cAMP production and cyclic nucleotide-gated (CNG) channel opening, raising intraciliary Ca^2+^ and amplifying the initial signal. Second, this Ca^2+^ influx activates transmembrane protein 16B (TMEM16B; also known as ANO2) Cl^−^ channels (Kleene and Gesteland, 1991; Kurahashi and Yau, 1993; Reisert et al., 2003). Because the Na^+^-K^+^-Cl^−^ cotransporter 1 (NKCC1; also known as SLC12A2) maintains high intraciliary Cl^−^ (∼50 mM versus ∼10 mM outside) (Kaneko et al., 2004), Cl^−^ efflux amplifies the initial current induced by the original odorant signal by nearly tenfold (Dibattista et al., 2017; Hengl et al., 2010; Mykytyn and Askwith, 2017). This two-step amplification ensures even small stimuli generate robust electrical responses, reliably depolarizing the neuron for accurate odor discrimination.

In rod outer segments, cGMP levels are high in the dark, which keeps CNG channels open. In mice, single photon detection leads to hydrolysis of ∼2000 cGMP molecules and CNG channel closure, halting the influx of ∼10^6^ positive ions across hundreds of channels, thus causing a voltage change (Pugh and Lamb, 1993; Rieke and Baylor, 1998). The astounding magnitude of amplification leverages both cilia compartmentalization and the small cilioplasmic volume.

Positive feedback loops are another form of amplification that can amplify signals, change signal response timing and create bistable switches (Brandman and Meyer, 2008). One notable positive feedback loop in sperm flagella involves the pH-sensitive soluble adenylyl cyclase (sAC) and the voltage- and cAMP-activated Na^+^/H^+^ exchanger (sNHE). H^+^ export alkalizes the cilioplasm, which stimulates sAC, boosting cAMP synthesis; in turn, rising cAMP levels increase sNHE sensitivity to voltage, further promoting H^+^ export (Körschen et al., 2021 preprint; Windler et al., 2018). This positive feedback creates a bistable switch that maintains sperm capacitation.

One type of amplification is notably absent from ciliary signaling: Ca^2+^-induced Ca^2+^ release, which opens ER Ca^2+^ channels driving all-or-nothing signaling responses. Because the ER is excluded from cilia, ions only enter the cilioplasm by diffusion from the base (following chemical gradients) or through channels in the ciliary membrane. Consequently, signaling within cilia is analog in nature, providing graded responses to signal detection.

Amplification in ciliary signaling both enables extreme sensitivity and reduces the impact of noise (see Glossary) from random fluctuations in pathway component concentration or localization. Amplification further increases the impact of true signals (Levine et al., 2007) and ensures reliable information transfer by creating high signal-to-noise ratios.

### Negative feedback and feedforward

Feedback and feedforward regulatory circuits are fundamental to cellular signaling, controlling the timing, amplitude and duration of pathway outputs and reducing the impact of noise (Alon, 2007). Although bow-tie network architecture commonly involves regulatory circuits, cilia signaling pathways are unique in that ciliary infrastructure and tuning mechanisms participate in these motifs. For example, the negative feedback mechanisms mentioned earlier that orchestrate ubiquitylated GPCR removal utilize retrograde IFT for export (Lange et al., 2025; Shinde et al., 2020; Ye et al., 2018). This limits the signaling window, thereby limiting pathway activation and promoting adaptation to persistent stimuli. Such feedback loops are fundamental for maintaining signaling homeostasis and responsiveness.

Negative feedback in the hedgehog pathway leverages ciliary compartmentalization – the SHH receptor PTCH1 can both alter the local lipid environment and directly remove sterols from SMO, a key activating effector, preventing SMO accumulation in cilia (Kinnebrew et al., 2019, 2021; Petrov et al., 2021). SHH binding causes PTCH1 internalization, which permits SMO accumulation in cilia and leads to activation of GLI family transcription factors. Active GLI promotes synthesis of new PTCH1, which returns to the cilium and inhibits SMO, turning off the pathway and restoring baseline activity (Hilgendorf et al., 2024). This cilia-based negative feedback prevents excessive or prolonged signaling and resets the pathway for future activation.

Ciliary length regulation also involves feedback. Pathways that drive changes in ciliary Ca^2+^ and cAMP, such as those involving somatostatin receptor type 3 (SSTR3) and other GPCRs that signal through Gα_s_ or Gα_i_, could alter AC3 activity and initiate feedback loops that modulate ciliary length (Besschetnova et al., 2010; Ou et al., 2009). Increased ciliary cAMP promotes elongation, whereas elevated cAMP in the cell body causes cilia shortening, reflecting spatially distinct negative feedback mediated by PKA (Hansen et al., 2020). Kinases in the v-ros cross-hybridizing kinase (RCK) family, such as ICK (also known as CILK1) and MAK, also sense ciliary length and adjust IFT speeds, and faster anterograde IFT promotes elongation whereas slower transport shortens cilia (Broekhuis et al., 2014). However, it is unclear whether length regulation generates positive or negative feedback (Wang and Ogden, 2025). Elongation could dilute signaling molecules by increasing volume; however, if receptor density is maintained, elongation could increase the probability of receptor–ligand encounters.

Feedforward loops are also a part of cilia regulation. A coherent feedforward loop occurs during pre-mitotic cilia disassembly; Aurora A kinase activates the deacetylase HDAC6, which both deacetylates tubulin to destabilize MTs and deacetylates the actin-binding protein cortactin to promote actin filament formation, reinforcing disassembly (Ran et al., 2015). We hypothesize that many ciliary signaling pathways leverage the small volume, high protein concentration and size-specific diffusion barriers of cilia to amplify the impact of feedforward modules compared to other cellular compartments, enabling tighter temporal control and enhanced signal-to-noise ratios.

### Crosstalk

The unique ciliary architecture creates a specialized environment that both shields against unwanted crosstalk with cytoplasmic pathways and enables selective interactions between ciliary signaling pathways (Hilgendorf et al., 2024; Marley et al., 2013). Crosstalk can occur at the receptor level or within the conserved processing core of the bow-tie network architecture, where pathways share core effectors or second messengers.

Cilia can insulate signaling pathways from crosstalk. Compartmentalization limits non-targeted protein entry, reducing promiscuous interactions and noise (Ladbury and Arold, 2012). High protein concentrations in cilia promote clustering and enzyme scaffold association, further minimizing interference and enhancing signal-to-noise ratio (Ladbury and Arold, 2012).

For receptors that localize both to the cell surface and to cilia, localization distribution regulates crosstalk. GPR88, for example, is a GPCR that functions both inside and outside cilia and its subcellular localization can influence signaling sensitivity and specificity. When GPR88 localizes to the cilium, it exhibits reduced crosstalk with signaling from the D1 dopamine receptor (Marley et al., 2013). By redistributing receptors, cells thereby fine-tune pathway specificity and sensitivity.

Cilia also insulate signaling from cytoplasmic fluctuations. For example, ER-mediated Ca^2+^ release is unlikely to impact cilia-confined signaling pathways. Likewise, PM receptors often undergo co-internalization in clathrin-coated pits (Alfonzo-Méndez et al., 2024), whereas ciliary receptors use distinct trafficking routes and remain shielded from this process.

Receptor-level crosstalk can occur through direct protein–protein interactions, such as heteromeric complexes, which can alter ligand binding or G protein coupling (Rozenfeld and Devi, 2010). High receptor concentrations can increase binding probability and promote complex formation. Additionally, tuning ciliary content might facilitate customized heteromeric pairing; for example, in neuronal cilia, the GPCRs MCHR1 and SSTR3 can form heteromeric complexes (Green et al., 2012). Heteromerization can also involve different receptor families (Liberto et al., 2019); for instance, the transforming growth factor-β (TGF-β) type I receptor (TβRI) can activate SMO when both are present in the cilium (Gencer et al., 2017). Coordinated IFT import could be a mechanism to promote heteromer formation by colocalizing receptors upon entry (Badgandi et al., 2017; Hesketh et al., 2022; Mukhopadhyay et al., 2010).

Heterologous desensitization (see Glossary) is an indirect form of receptor crosstalk. Cilia receptor activation leads to β-arrestin recruitment, ubiquitylation and BBSome-mediated removal by IFT (described above). This process can also internalize unrelated receptors that are indiscriminately ubiquitylated by β-arrestin. For example, SMO activation leads to β-arrestin-dependent removal of GPR161 from cilia (Pal et al., 2016), a process also proposed for other cilia signaling pathways (Wang and Ogden, 2025).

Effector-level crosstalk is common in cilia, as many pathways share second messengers and core effectors. Cross-activation occurs when one pathway directly activates another; for example, SSTR3 activation can induce nuclear Gli2 accumulation because both pathways share adenylyl cyclase and PKA (Nilsson et al., 2025). Cross-inhibition has been shown using an engineered light-activated GPCR that raises cAMP, promotes Gli3 cleavage and represses SHH signaling (Truong et al., 2021).

Effector competition (see Glossary), although related, is distinct from cross-inhibition. It occurs when multiple pathways vie for access to a limiting pool of shared effector (Brinkerhoff et al., 2008). In cilia, PKA is a downstream effector for GPR161 and several other GPCRs, but GPR161 has a unique advantage – a dedicated PKA-binding domain (Mukhopadhyay et al., 2013), which enables it to sequester PKA. In this way, GPR161 limits PKA availability for other ciliary receptors.

Synergistic crosstalk occurs when multiple pathways converge on shared effectors to amplify the signaling output. In multiciliated cells, ATP and adenosine together increase the beat frequency of cilia more strongly than either alone (Morales et al., 2000). Such synergy enables coincidence detection, ensuring responses occur only when multiple signals are present.

In summary, the spatial constraints and biochemical environment of cilia create a signaling compartment where pathway crosstalk is insulated from events in the cell body. By selectively localizing signaling proteins to cilia, the nature and extent of ciliary crosstalk can be precisely controlled, enabling highly specific and context-dependent signaling outcomes. Together, the design elements – amplification, feedforward and feedback loops and crosstalk – are essential for cilia signaling, each creating emergent properties that change the magnitude, frequency and temporal dynamics when integrated into signaling networks.

### Pathway level sensitivity

Pathway sensitivity depends on how individual reactions and network motifs assemble into higher-order architectures. Although olfaction, vision and flagellar chemosensing have been well-characterized, most ciliary signaling pathways have not. Other pathway designs might use ciliary compartmentalization and high local concentrations to create distinct sensitivity profiles. Below, we highlight pathway strategies that could leverage unique ciliary features.

Sperm flagella and photoreceptors use extreme receptor density to achieve exquisite sensitivity. Another way to promote sensitivity at low ligand concentrations is through receptor reserve (see Glossary; Hoyer and Boddeke, 1993; Zhu, 1993). Although typically studied in pharmacological contexts and not yet demonstrated in cilia, receptor reserve is a plausible sensitivity tuning mechanism. For example, if a ciliary GPCR greatly outnumbers available G proteins, maximal activation could occur at ligand concentrations well below the *K*_D_ for the receptor. Receptors such as GPR88, which can localize to either cilia or the PM (Marley et al., 2013), might reach a full response in the ciliary pool at a lower concentration than the PM pool if ciliary effectors are limiting. Artificially created receptor reserve, generated, for example, through ciliary receptor overexpression, could shift pathway sensitivity and complicate data interpretation.

Pathway configurations that are ultrasensitive (see Glossary) switch rapidly on or off at threshold concentrations, similar to cooperative ligand binding (Goldbeter and Koshland, 1981). We hypothesize that the high concentrations achievable in cilia with few molecules could promote ultrasensitive signaling. Cilia could facilitate multi-step ultrasensitivity (see Glossary) by isolating and concentrating proteins and scaffolding enzymes. Alternately, saturating concentrations of small molecules or other substrates in cilia could contribute to zero-order ultrasensitivity (see Glossary) in pathways in which opposing zero-order reactions (e.g. adenylyl cyclase and phosphodiesterase) are present. In this case, changes in enzyme concentration would regulate pathway activity (Ferrell and Ha, 2014a). The ability of primary cilia to selectively concentrate proteins, lipids, ions and cyclic nucleotides underlies these possible signaling regimes.

## Robustness in cilia

In addition to sensitivity, robustness is an important attribute for signal transduction. Pathways must maintain function despite perturbations such as changes in ligand properties, protein abundance or random mutations. Key determinants of robustness include redundancy, modularity, decoupling and system control (Kitano, 2004). In cilia, these strategies are embedded across both their physical structure and network architecture to support stable signal transmission under varied conditions.

At the structural level, robustness ensures that core functions are preserved under perturbation. In cilia, the nine-fold MT symmetry in the centriole and proximal cilium provides redundancy, and the TZ is reinforced by repeating structures rather than a single collar. Both the MTs and Y-links demonstrate modularity (Park and Leroux, 2022). The IFT system is also modular, consisting of laterally associating complexes (‘trains’) (Lacey and Pigino, 2025). Decoupling is achieved by the isolation of the cilioplasm and ciliary membrane – only specifically trafficked proteins can access these compartments. System controls further coordinate ciliary dimensions, including length, volume and surface area, to maintain integrity.

At the biochemical level, robustness means that reaction outcomes remain stable despite fluctuations (Barkai and Leibler, 1997). At the individual reaction level, it is possible that substrate saturation promotes robustness by reducing sensitivity to concentration changes. Network elements such as amplification, feedback, feedforward and noise management can each be robust if they function despite variations in input or parameter values. Compartmentalization enhances robustness by insulating reactions from extrinsic noise and enabling a controlled environment. These robust elements form the foundation for higher-order pathway robustness.

At the pathway level, robustness refers to the capacity of a signaling pathway to transmit information reliably despite disturbances or environmental changes. Although many pathways achieve this through modularity and redundancy, cilia are unique in their ability to decouple signaling from the rest of the cell.

System-level controls, which include network design elements, are central to robustness. Negative feedback mechanisms buffer variation and stabilize pathway output, sometimes trading off with sensitivity (Blüthgen and Legewie, 2013). Feedforward loops add redundancy by providing alternative routes for signal propagation. Together, these design features enable effective noise management, stabilizing signaling during stochastic fluctuations or perturbations in component abundance (Ladbury and Arold, 2012).

Weak linkage (see Glossary) between reactions in the processing layer is essential for bow-tie architecture robustness (Kitano, 2004). In cilia, weak linkage is exemplified by diffusible messengers, such as cAMP and Ca^2+^, which flexibly engage multiple effectors. This flexibility not only prevents complete pathway failure but also enables evolutionary innovation, as new components can interface with existing machinery without requiring complete system redesign (Kirschner and Gerhart, 1998).

Network motifs such as feedforward and feedback loops further strengthen bow-tie architectures by providing alternative transmission routes and adaptive, stable responses. In RTK signaling, another bow-tie system, feedback loops from receptor and output layers act on longer timescales than those within the processing layer (Lemmon et al., 2016) – a pattern likely shared by ciliary signaling. For example, feedback affecting receptor internalization, cilia length or transcription occurs more slowly than feedback affecting effector modification or second messenger concentrations.

The combination of bow-tie signaling logic and specialized ciliary architecture makes ciliary signaling distinct from other cellular pathways. Unlike more diffusely organized networks, ciliary signaling depends on spatial concentration and core effector tuning within an isolated organelle. Yet, the very features that underpin robustness also create potential vulnerabilities, or fragile points (see Glossary), raising the question of how ciliary systems fail and what this reveals about cilia-related pathologies.

## Fragile points and ciliopathies

The very structures that confer robustness can also create fragile points susceptible to rare, unanticipated disruptions. In cilia, robustness depends on structural integrity, selective permeability barrier maintenance and precise protein trafficking. Disruption of any of these compromises ciliary function and leads to disease (Reiter and Leroux, 2017).

The IFT system represents a fundamental fragile point. IFT is essential for cilium assembly, maintenance and cargo trafficking, with core components (e.g. the KIF3A motor subunit and the IFT-B complex protein IFT88) exhibiting near-zero redundancy. Global disruption of *Kif3a* or *Ift88* causes embryonic lethality in mice due to cilia absence and SHH signaling failure, underscoring the non-redundant role of IFT as a systemic fragile point (Eggenschwiler and Anderson, 2007). In humans, mutations in IFT components cause severe developmental and physiological defects (Reiter and Leroux, 2017). This fragility has also been exploited experimentally – conditional disruption of IFT genes using Cre-lox systems enables tissue-specific or temporal cilia ablation, revealing diverse roles in organ development, signaling and disease (Davenport et al., 2007; Haycraft et al., 2007).

Additional fragile points have been revealed through the study of ciliopathies – genetic disorders caused by mutations in components affecting ciliary formation or function. These include the centrosome, axoneme, TZ, centriolar satellites, the inversin subcompartment and trafficking machinery. Among these, the TZ stands out as a particularly crucial node (Reiter and Leroux, 2017). Mutations in TZ components are responsible for a spectrum of severe ciliopathies, such as Meckel syndrome, Joubert syndrome, nephronophthisis, Senior–Løken syndrome, oral-facial-digital syndrome and Leber congenital amaurosis (Reiter and Leroux, 2017).

The clinical phenotype diversity associated with TZ defects – ranging from kidney cysts to neurological and skeletal abnormalities (Mill et al., 2023) – raises the question of why disruption of a single structural module produces a wide array of diseases. The answer lies in the modular and decoupled architecture of the TZ, which gives it robust features and distinct vulnerabilities. The MKS complex, located closest to the membrane, regulates membrane gating, and the NPHP complex, which encircles the axoneme, anchors the TZ to MTs (Shi et al., 2017; Yang et al., 2015). Disease-causing mutations might disrupt a single module without abolishing all cilia functions (Shi et al., 2017).

If the TZ is robust, where are its fragile points? As with cilia overall, the features that confer robustness – especially modularity and decoupling – can concentrate fragility at crucial connecting nodes (Csete and Doyle, 2004). Centrosome protein CEP290 is one example: as a large scaffold protein that links distinct TZ modules to the centriole and contributes to the ciliary gate, CEP290 integrates otherwise decoupled components (Park and Leroux, 2022). Because it is highly connected and functionally irreplaceable, disruptions to CEP290 can uncouple multiple modules. Notably, different mutations in CEP290 can disrupt specific module connections to varying degrees (Park and Leroux, 2022), which might explain why CEP290 mutations cause a heterogeneous range of ciliopathy phenotypes.

Cilium robustness depends on TZ robustness. Decoupling and modularity enable partial function even when fragile points are disrupted – some signaling pathways or structural features can persist. However, understanding how individual mutations affect underlying module function and connectivity could explain why some ciliopathies are syndromic and severe, whereas others are tissue-restricted or variable.

The ciliary bow-tie network architecture, although robust, also creates fragile points (Csete and Doyle, 2004; Kitano, 2004). Mutations in the input layer typically affect individual pathways (such as SHH or polycystin). In contrast, mutations that disrupt the core network architecture have broad effects. For example, the BBSome is essential for negative feedback between the processing and receptor layers. Mutations that disable IFT loading of the BBSome disrupt this network architecture, making the BBSome a fragile point that affects activated GPCR trafficking.

The robust-yet-fragile nature of cilia provides insight into how disruptions cause disease and illuminates a fundamental biological trade-off – complex architectures must optimize for current function while maintaining the capacity for evolutionary innovation (Carlson and Doyle, 2002; Csete and Doyle, 2004; Kitano, 2004). Understanding how evolution balances this trade-off reveals a previously unexplored feature of cilia: evolvability.

## Evolvability of cilia

Evolvability (see Glossary) is the ability to generate heritable variation that produces new traits. Because it enhances both current fitness and future adaptability, evolvability can itself be a target of natural selection (Kirschner and Gerhart, 1998). Several features contribute to evolvability – versatile protein elements, weak linkage, exploratory mechanisms, compartmentalization and redundancy – all of which also support robustness and flexibility (Kirschner and Gerhart, 1998; Kitano, 2004). Weak linkages and versatile elements, central to the bow-tie network architecture, allow signaling pathways to converge diverse receptors onto conserved core processes, thereby promoting both robustness and evolvability (Csete and Doyle, 2004; Kitano, 2004).

The deep evolutionary conservation of cilia across eukaryotes raises two questions – why have cilia been conserved, and how do they support such diverse signaling pathways? We propose the answer lies in their bow-tie network architecture anchored to the robust ciliary physical structure, which collectively make cilia highly evolvable.

Both evolvability and robustness depend on balancing flexibility with conserving core processes. Evolutionarily, this balance involves constraint and deconstraint (see Glossary). Structural variations in sensory cilia illustrate this balance between evolutionary flexibility and conserved core machinery. In photoreceptors, rods and cones display distinct structural innovations; rods contain stacked membrane disks, whereas cones form continuous membrane infoldings, with both adaptations increasing the surface area for phototransduction. Another adaptation can be changes in cilia number – most cell types feature a single cilium, but olfactory sensory neurons possess multiple cilia, enhancing odor detection (Menco and Ph, 1997). Likewise, certain hypothalamic neurons (GnRH neurons) extend multiple cilia during puberty (Koemeter-Cox et al., 2014). Cilia length also varies across cell types and developmental stages, perhaps responding to specialized signaling requirements (Ishikawa and Marshall, 2011). Despite this diversity, the underlying ciliary axoneme, TZ and IFT machinery remain highly conserved, providing a robust core that supports the evolution of specialized functions.

Cilia exhibit structural flexibility like a skyscraper that is reinforced at its base yet is flexible in its upper floors. The centriole constrains MTs at the base, where doublet MTs and MT-binding proteins provide stability, whereas toward the distal cilium, fewer MT-binding proteins and individual doublets can terminate or transition to singlets, providing flexibility (Gluenz et al., 2010; Kiesel et al., 2020; Ott et al., 2024b).

Deconstraint is also evident in tissue-specific adaptations; for example, TZs in astrocytes in the visual cortex are ∼100 nm shorter than those in neurons (Ott et al., 2024b). Such differences likely reflect cell type-specific protein expression, isoforms and splice variants (Garcia-Gonzalo et al., 2011; Jana et al., 2018; Yang et al., 2015). Although this specialization empowers cilia to adjust to diverse cellular contexts, it also means certain mutations are buffered by redundancy, restricting dysfunction to particular cell types (Kitano, 2004).

The bow-tie architecture in cilia also balances constraint and deconstraint. Conserved core machinery constrains the system by maintaining essential signaling, but simultaneously deconstrains it by offering versatile elements – such as G proteins, cAMP, Ca^2+^ and ion channels – that can interact with diverse receptors and effectors (Csete and Doyle, 2004; Kitano, 2004). Weak linkages in the processing layer reduce interdependence, so individual pathway elements can change without disrupting the system (Csete and Doyle, 2004; Kirschner and Gerhart, 1998; Kitano, 2004). This flexibility has enabled different GPCRs, RTKs and other receptors to be added to the input layer, and transcription factors, kinases and other effectors to the output layer, without redesigning core machinery. This flexibility explains how the network architecture can support diverse signaling pathways.

Because all bow-tie architectures are inherently evolvable (Csete and Doyle, 2004; Kitano, 2004), integrating this architecture into the cilium confers unique advantages. The versatile elements and weak linkages inside cilia lower the number of mutations required for phenotypic innovation. Simultaneously, because the receptor and processing layers are isolated, new signaling combinations can be explored without endangering essential cellular functions. This reduces the chances that evolutionary exploration – genetic variation followed by selection – will become lethal.

Together, bow-tie network organization and ciliary structure and compartmentalization provide both the robustness required for survival alongside the adaptability needed for evolution. This dual architecture explains why cilia have been conserved across eukaryotes and how they can accommodate such extensive signaling pathway diversity.

## Conclusions

Cilia geometry and infrastructure establish a specialized environment for signaling. Because nearly all downstream events triggered by cilia-localized receptors occur within this organelle, the ciliary microenvironment shapes the magnitude, timing and character of each step in every ciliary signaling pathway. Cilia have been conserved throughout evolution because their structural and processing machinery provides a robust framework for signaling detection and transmission. At the same time, flexibility in input and output layers allows adaptability, explaining both the diversification and evolutionary conservation of cilia.

The unique properties of cilia influence signaling not only within individual cells but also across cell populations. Ciliary protein composition varies both within and between cell populations (Green et al., 2012; Hansen et al., 2025). Whereas the signaling profile of an individual cell reflects its distinct receptor repertoire, population-level diversity expands the collective capacity for information processing. This distributed architecture enables broader sensing, enhanced signal integration and coordinated responses across tissues. For example, photoreceptors rely on rod–cone diversity and distinct adaptation mechanisms – rods adjust their operating range through light adaptation, whereas cones tune their dynamic range according to wavelength at the ciliary level (Yau and Hardie, 2009). Such organelle- and population-scale diversity allows tissues to generate nuanced and robust responses to environmental cues.

Notably, not all cell types retain cilia (Ott and Mukhopadhyay, 2025). In mixed cell populations, heterogeneity might be essential for tuning responsiveness (Ott and Mukhopadhyay, 2025). For example, non-ciliated cells can ignore proliferative cues sensed by neighboring ciliated cells, as observed in adipose tissue and cerebellar granule cell neurons (Constable et al., 2024; Hilgendorf et al., 2019; Ott et al., 2024a). Moreover, population-level shifts in cilia frequency, such as those occurring during circadian modulation in the suprachiasmatic nucleus, might coordinate tissue-wide signaling responses (Tu et al., 2023). Understanding how ciliary signaling contributes to collective cellular behavior remains an important frontier.

Although this discussion has emphasized primary cilia and chemosensory sperm flagella, motile cilia also function as sensory organelles (Bloodgood, 2010; Shah et al., 2009), although much less is known about signaling in these cilia. Like primary cilia, motile cilia rely on selective trafficking and local concentration of signaling molecules, with additional structural features supporting motility. Although motility does not improve signal detection in uniform environments (Berg and Purcell, 1977), the increased surface area of multiciliated cells can expand sampling capacity and amplify downstream signaling output.

The ideas proposed here are grounded in decades of research on proximate questions including structural analyses, concentration measurements of small molecules, identification of cilia-localized receptors, and studies of ciliary trafficking and targeting. These foundational insights have set the stage for addressing ultimate questions concerning the physiological and evolutionary significance of cilia. Nonetheless, more experimental data are needed to fully elucidate individual pathway mechanisms and explore predictions emerging from these theories. Several pressing questions remain. Why do mutations in different ciliary components give rise to distinct syndromes? Why do ciliopathies display such striking clinical heterogeneity, even among individuals carrying similar mutations in the same protein? How do genetic alterations impact signaling tunability, sensitivity and robustness across reactions, pathways, networks and populations of cells? Addressing these questions will not only clarify the role of cilia in evolution and physiology but also guide new strategies for diagnosing, treating and ultimately preventing cilia-associated diseases.

## References

[JCS264325C1] Adam, G. and Delbrück, M. (1968). Reduction of dimensionality in biological diffusion processes. In *Structural Chemistry and Molecular Biology* (ed. A. Rich and N. Davidson), pp. 198-215. W. H. Freeman and Company.

[JCS264325C2] Alfonzo-Méndez, M. A., Strub, M.-P. and Taraska, J. W. (2024). Spatial and signaling overlap of growth factor receptor systems at clathrin-coated sites. *Mol. Biol. Cell*, 35, ar138. 10.1091/mbc.E24-05-0226 39292879 PMC11617105

[JCS264325C3] Alon, U. (2007). Network motifs: theory and experimental approaches. *Nat. Rev. Genet.* 8, 450-461. 10.1038/nrg2102 17510665

[JCS264325C4] Anvarian, Z., Mykytyn, K., Mukhopadhyay, S., Pedersen, L. B. and Christensen, S. T. (2019). Cellular signalling by primary cilia in development, organ function and disease. *Nat. Rev. Nephrol.* 15, 199-219. 10.1038/s41581-019-0116-9 30733609 PMC6426138

[JCS264325C5] Badgandi, H. B., Hwang, S., Shimada, I. S., Loriot, E. and Mukhopadhyay, S. (2017). Tubby family proteins are adapters for ciliary trafficking of integral membrane proteins. *J. Cell Biol.* 216, 743-760. 10.1083/jcb.201607095 28154160 PMC5350516

[JCS264325C6] Barabási, A.-L. and Oltvai, Z. N. (2004). Network biology: understanding the cell's functional organization. *Nat. Rev. Genet.* 5, 101-113. 10.1038/nrg1272 14735121

[JCS264325C7] Barkai, N. and Leibler, S. (1997). Robustness in simple biochemical networks. *Nature* 387, 913-917. 10.1038/43199 9202124

[JCS264325C8] Berg, H. C. and Purcell, E. M. (1977). Physics of chemoreception. *Biophys. J.* 20, 193-219. 10.1016/S0006-3495(77)85544-6 911982 PMC1473391

[JCS264325C9] Bergman, T. J. and Beehner, J. C. (2022). Leveling with Tinbergen: Four levels simplified to causes and consequences. *Evol. Anthropol.* 31, 12-19. 10.1002/evan.21931 34904769

[JCS264325C10] Bernard, A., Naharros, I. O., Yue, X., Mifsud, F., Blake, A., Bourgain-Guglielmetti, F., Ciprin, J., Zhang, S., McDaid, E., Kim, K. et al. (2023). MRAP2 regulates energy homeostasis by promoting primary cilia localization of MC4R. *JCI Insight* 8, e155900. 10.1172/jci.insight.155900 36692018 PMC9977312

[JCS264325C11] Besschetnova, T. Y., Kolpakova-Hart, E., Guan, Y., Zhou, J., Olsen, B. R. and Shah, J. V. (2010). Identification of signaling pathways regulating primary cilium length and flow-mediated adaptation. *Curr. Biol.* 20, 182-187. 10.1016/j.cub.2009.11.072 20096584 PMC2990526

[JCS264325C12] Binggeli, R. and Weinstein, R. C. (1986). Membrane potentials and sodium channels: hypotheses for growth regulation and cancer formation based on changes in sodium channels and gap junctions. *J. Theor. Biol.* 123, 377-401. 10.1016/S0022-5193(86)80209-0 2443763

[JCS264325C13] Bishop, G. A., Berbari, N. F., Lewis, J. and Mykytyn, K. (2007). Type III adenylyl cyclase localizes to primary cilia throughout the adult mouse brain. *J. Comp. Neurol.* 505, 562-571. 10.1002/cne.21510 17924533

[JCS264325C14] Bloodgood, R. A. (2010). Sensory reception is an attribute of both primary cilia and motile cilia. *J. Cell Sci.* 123, 505-509. 10.1242/jcs.066308 20144998

[JCS264325C15] Blüthgen, N. and Legewie, S. (2013). Robustness of signal transduction pathways. *Cell. Mol. Life Sci.* 70, 2259-2269. 10.1007/s00018-012-1162-7 23007845 PMC11113274

[JCS264325C16] Brandman, O. and Meyer, T. (2008). Feedback loops shape cellular signals in space and time. *Science* 322, 390-395. 10.1126/science.1160617 18927383 PMC2680159

[JCS264325C17] Brewer, K. M., Brewer, K. K., Richardson, N. C. and Berbari, N. F. (2022). Neuronal cilia in energy homeostasis. *Front. Cell Dev. Biol.* 10, 1082141. 10.3389/fcell.2022.1082141 36568981 PMC9773564

[JCS264325C18] Brinkerhoff, C. J., Traynor, J. R. and Linderman, J. J. (2008). Collision coupling, crosstalk, and compartmentalization in G-protein coupled receptor systems: can a single model explain disparate results? *J. Theor. Biol.* 255, 278-286. 10.1016/j.jtbi.2008.08.003 18761019 PMC2917770

[JCS264325C19] Broekhuis, J. R., Verhey, K. J. and Jansen, G. (2014). Regulation of cilium length and intraflagellar transport by the RCK-kinases ICK and MOK in renal epithelial cells. *PLoS ONE* 9, e108470. 10.1371/journal.pone.0108470 25243405 PMC4171540

[JCS264325C20] Carlson, J. M. and Doyle, J. (2002). Complexity and robustness. *Proc. Natl. Acad. Sci. USA* 99, 2538-2545. 10.1073/pnas.012582499 11875207 PMC128573

[JCS264325C21] Choi, Y.-H., Suzuki, A., Hajarnis, S., Ma, Z., Chapin, H. C., Caplan, M. J., Pontoglio, M., Somlo, S. and Igarashi, P. (2011). Polycystin-2 and phosphodiesterase 4C are components of a ciliary A-kinase anchoring protein complex that is disrupted in cystic kidney diseases. *Proc. Natl. Acad. Sci. USA* 108, 10679-10684. 10.1073/pnas.1016214108 21670265 PMC3127890

[JCS264325C22] Conduit, S. E., Davies, E. M., Fulcher, A. J., Oorschot, V. and Mitchell, C. A. (2021). superresolution microscopy reveals distinct phosphoinositide subdomains within the cilia transition zone. *Front. Cell Dev. Biol.* 9, 634649. 10.3389/fcell.2021.634649 33996795 PMC8120242

[JCS264325C23] Constable, S., Ott, C. M., Lemire, A. L., White, K., Xun, Y., Lim, A., Lippincott-Schwartz, J. and Mukhopadhyay, S. (2024). Permanent cilia loss during cerebellar granule cell neurogenesis involves withdrawal of cilia maintenance and centriole capping. *Proc. Natl. Acad. Sci. USA* 121, e2408083121. 10.1073/pnas.2408083121 39705308 PMC11670249

[JCS264325C24] Csete, M. and Doyle, J. (2004). Bow ties, metabolism and disease. *Trends Biotechnol.* 22, 446-450. 10.1016/j.tibtech.2004.07.007 15331224

[JCS264325C25] Davenport, J. R., Watts, A. J., Roper, V. C., Croyle, M. J., van Groen, T., Wyss, J. M., Nagy, T. R., Kesterson, R. A. and Yoder, B. K. (2007). Disruption of intraflagellar transport in adult mice leads to obesity and slow-onset cystic kidney disease. *Curr. Biol.* 17, 1586-1594. 10.1016/j.cub.2007.08.034 17825558 PMC2084209

[JCS264325C26] DeCaen, P. G., Delling, M., Vien, T. N. and Clapham, D. E. (2013). Direct recording and molecular identification of the calcium channel of primary cilia. *Nature* 504, 315-318. 10.1038/nature12832 24336289 PMC4073646

[JCS264325C27] Delling, M., DeCaen, P. G., Doerner, J. F., Febvay, S. and Clapham, D. E. (2013). Primary cilia are specialized calcium signalling organelles. *Nature* 504, 311-314. 10.1038/nature12833 24336288 PMC4112737

[JCS264325C28] Derderian, C., Canales, G. I. and Reiter, J. F. (2023). Seriously cilia: a tiny organelle illuminates evolution, disease, and intercellular communication. *Dev. Cell* 58, 1333-1349. 10.1016/j.devcel.2023.06.013 37490910 PMC10880727

[JCS264325C29] Dibattista, M., Pifferi, S., Boccaccio, A., Menini, A. and Reisert, J. (2017). The long tale of the calcium activated Cl− channels in olfactory transduction. *Channels* 11, 399-414. 10.1080/19336950.2017.1307489 28301269 PMC5626357

[JCS264325C30] Eggenschwiler, J. T. and Anderson, K. V. (2007). Cilia and developmental signaling. *Annu. Rev. Cell Dev. Biol.* 23, 345-373. 10.1146/annurev.cellbio.23.090506.123249 17506691 PMC2094042

[JCS264325C31] Eisner, D., Neher, E., Taschenberger, H. and Smith, G. (2023). Physiology of intracellular calcium buffering. *Physiol. Rev.* 103, 2767-2845. 10.1152/physrev.00042.2022 37326298 PMC11550887

[JCS264325C32] Ferrell, J. E. (2002). Self-perpetuating states in signal transduction: positive feedback, double-negative feedback and bistability. *Curr. Opin. Cell Biol.* 14, 140-148. 10.1016/S0955-0674(02)00314-9 11891111

[JCS264325C33] Ferrell, J. E. and Ha, S. H. (2014a). Ultrasensitivity part I: Michaelian responses and zero-order ultrasensitivity. *Trends Biochem. Sci.* 39, 496-503. 10.1016/j.tibs.2014.08.003 25240485 PMC4214216

[JCS264325C34] Ferrell, J. E. and Ha, S. H. (2014b). Ultrasensitivity part III: cascades, bistable switches, and oscillators. *Trends Biochem. Sci.* 39, 612-618. 10.1016/j.tibs.2014.10.002 25456048 PMC4254632

[JCS264325C35] Ferrell, J. E., Jr and Ha, S. H. (2014). Ultrasensitivity part II: multisite phosphorylation, stoichiometric inhibitors, and positive feedback. *Trends Biochem. Sci.* 39, 556-569. 10.1016/j.tibs.2014.09.003 25440716 PMC4435807

[JCS264325C36] Garcia-Gonzalo, F. R. and Reiter, J. F. (2017). Open sesame: how transition fibers and the transition zone control ciliary composition. *Cold Spring Harb. Perspect. Biol.* 9, a028134. 10.1101/cshperspect.a028134 27770015 PMC5287074

[JCS264325C37] Garcia-Gonzalo, F. R., Corbit, K. C., Sirerol-Piquer, M. S., Ramaswami, G., Otto, E. A., Noriega, T. R., Seol, A. D., Robinson, J. F., Bennett, C. L., Josifova, D. J. et al. (2011). A transition zone complex regulates mammalian ciliogenesis and ciliary membrane composition. *Nat. Genet.* 43, 776-784. 10.1038/ng.891 21725307 PMC3145011

[JCS264325C38] Garcia-Gonzalo, F. R., Phua, S. C., Roberson, E. C., Garcia, G., Abedin, M., Schurmans, S., Inoue, T. and Reiter, J. F. (2015). Phosphoinositides regulate ciliary protein trafficking to modulate hedgehog signaling. *Dev. Cell* 34, 400-409. 10.1016/j.devcel.2015.08.001 26305592 PMC4557815

[JCS264325C39] Gencer, S., Oleinik, N., Kim, J., Selvam, S. P., Palma, R. D., Dany, M., Nganga, R., Thomas, R. J., Senkal, C. E., Howe, P. H. et al. (2017). TGF-β receptor I/II trafficking and signaling at primary cilia are inhibited by ceramide to attenuate cell migration and tumor metastasis. *Sci. Signal.* 10, eaam7464. 10.1126/scisignal.aam7464 29066540 PMC5818989

[JCS264325C40] Girshovitz, P. and Shaked, N. T. (2012). Generalized cell morphological parameters based on interferometric phase microscopy and their application to cell life cycle characterization. *Biomed. Opt. Express* 3, 1757-1773. 10.1364/BOE.3.001757 22876342 PMC3409697

[JCS264325C41] Gluenz, E., Höög, J. L., Smith, A. E., Dawe, H. R., Shaw, M. K. and Gull, K. (2010). Beyond 9+0: noncanonical axoneme structures characterize sensory cilia from protists to humans. *FASEB J.* 24, 3117-3121. 10.1096/fj.09-151381 20371625 PMC2923350

[JCS264325C42] Goentoro, L., Shoval, O., Kirschner, M. W. and Alon, U. (2009). The incoherent feedforward loop can provide fold-change detection in gene regulation. *Mol. Cell* 36, 894-899. 10.1016/j.molcel.2009.11.018 20005851 PMC2896310

[JCS264325C43] Goetz, S. C. and Anderson, K. V. (2010). The primary cilium: a signalling centre during vertebrate development. *Nat. Rev. Genet.* 11, 331-344. 10.1038/nrg2774 20395968 PMC3121168

[JCS264325C44] Goldbeter, A. and Koshland, D. E. (1981). An amplified sensitivity arising from covalent modification in biological systems. *Proc. Natl. Acad. Sci. USA* 78, 6840-6844. 10.1073/pnas.78.11.6840 6947258 PMC349147

[JCS264325C45] Green, J. A., Gu, C. and Mykytyn, K. (2012). Heteromerization of ciliary G protein-coupled receptors in the mouse brain. *PLoS ONE* 7, e46304. 10.1371/journal.pone.0046304 23029470 PMC3459911

[JCS264325C46] Guo, J., Otis, J. M., Higginbotham, H., Monckton, C., Cheng, J., Asokan, A., Mykytyn, K., Caspary, T., Stuber, G. D. and Anton, E. S. (2017). Primary Cilia signaling shapes the development of interneuronal connectivity. *Dev. Cell* 42, 286-300.e4. 10.1016/j.devcel.2017.07.010 28787594 PMC5571900

[JCS264325C47] Halls, M. L. and Cooper, D. M. F. (2011). Regulation by Ca2+-signaling pathways of adenylyl cyclases. *Cold Spring Harb. Perspect. Biol.* 3, a004143. 10.1101/cshperspect.a004143 21123395 PMC3003456

[JCS264325C48] Hansen, J. N., Kaiser, F., Klausen, C., Stüven, B., Chong, R., Bönigk, W., Mick, D. U., Möglich, A., Jurisch-Yaksi, N., Schmidt, F. I. et al. (2020). Nanobody-directed targeting of optogenetic tools to study signaling in the primary cilium. *eLife* 9, e57907. 10.7554/eLife.57907 32579112 PMC7338050

[JCS264325C49] Hansen, J. N., Sun, H., Kahnert, K., Westenius, E., Johannesson, A., Villegas, C., Le, T., Tzavlaki, K., Winsnes, C., Pohjanen, E. et al. (2025). Intrinsic heterogeneity of primary cilia revealed through spatial proteomics. *Cell*, S0092-8674(25)01029-3. 10.1016/j.cell.2025.08.039 41005307

[JCS264325C50] Haycraft, C. J., Zhang, Q., Song, B., Jackson, W. S., Detloff, P. J., Serra, R. and Yoder, B. K. (2007). Intraflagellar transport is essential for endochondral bone formation. *Development* 134, 307-316. 10.1242/dev.02732 17166921

[JCS264325C51] Hengl, T., Kaneko, H., Dauner, K., Vocke, K., Frings, S. and Möhrlen, F. (2010). Molecular components of signal amplification in olfactory sensory cilia. *Proc. Natl. Acad. Sci. USA* 107, 6052-6057. 10.1073/pnas.0909032107 20231443 PMC2851919

[JCS264325C52] Hesketh, S. J., Mukhopadhyay, A. G., Nakamura, D., Toropova, K. and Roberts, A. J. (2022). IFT-A structure reveals carriages for membrane protein transport into cilia. *Cell* 185, 4971-4985.e16. 10.1016/j.cell.2022.11.010 36462505

[JCS264325C53] Hilgendorf, K. I., Johnson, C. T., Mezger, A., Rice, S. L., Norris, A. M., Demeter, J., Greenleaf, W. J., Reiter, J. F., Kopinke, D. and Jackson, P. K. (2019). Omega-3 fatty acids activate ciliary FFAR4 to control adipogenesis. *Cell*. 179, 1289-1305.e21. 10.1016/j.cell.2019.11.005 31761534 PMC7332222

[JCS264325C54] Hilgendorf, K. I., Myers, B. R. and Reiter, J. F. (2024). Emerging mechanistic understanding of cilia function in cellular signalling. *Nat. Rev. Mol. Cell Biol.* 25, 555-573. 10.1038/s41580-023-00698-5 38366037 PMC11199107

[JCS264325C55] Hoyer, D. and Boddeke, H. W. G. M. (1993). Partial agonists, full agonists, antagonists: dilemmas of definition. *Trends Pharmacol. Sci.* 14, 270-275. 10.1016/0165-6147(93)90129-8 8105597

[JCS264325C56] Hu, Q., Milenkovic, L., Jin, H., Scott, M. P., Nachury, M. V., Spiliotis, E. T. and Nelson, W. J. (2010). A septin diffusion barrier at the base of the primary cilium maintains ciliary membrane protein distribution. *Science* 329, 436-439. 10.1126/science.1191054 20558667 PMC3092790

[JCS264325C57] Huangfu, D., Liu, A., Rakeman, A. S., Murcia, N. S., Niswander, L. and Anderson, K. V. (2003). Hedgehog signalling in the mouse requires intraflagellar transport proteins. *Nature* 426, 83-87. 10.1038/nature02061 14603322

[JCS264325C58] Hwang, S.-H., Somatilaka, B. N., White, K. and Mukhopadhyay, S. (2021). Ciliary and extraciliary Gpr161 pools repress hedgehog signaling in a tissue-specific manner. *eLife* 10, e67121. 10.7554/eLife.67121 34346313 PMC8378848

[JCS264325C59] Ishikawa, H. and Marshall, W. F. (2011). Ciliogenesis: building the cell's antenna. *Nat. Rev. Mol. Cell Biol.* 12, 222-234. 10.1038/nrm3085 21427764

[JCS264325C60] Jana, S. C., Mendonça, S., Machado, P., Werner, S., Rocha, J., Pereira, A., Maiato, H. and Bettencourt-Dias, M. (2018). Differential regulation of transition zone and centriole proteins contributes to ciliary base diversity. *Nat. Cell Biol.* 20, 928-941. 10.1038/s41556-018-0132-1 30013109

[JCS264325C61] Jiang, J. Y., Falcone, J. L., Curci, S. and Hofer, A. M. (2019). Direct visualization of cAMP signaling in primary cilia reveals up-regulation of ciliary GPCR activity following Hedgehog activation. *Proc. Natl. Acad. Sci. USA* 116, 12066-12071. 10.1073/pnas.1819730116 31142652 PMC6575585

[JCS264325C62] Johnson, C. A. and Malicki, J. J. (2019). The nuclear arsenal of Cilia. *Dev. Cell* 49, 161-170. 10.1016/j.devcel.2019.03.009 31014478

[JCS264325C63] Kaneko, H., Putzier, I., Frings, S., Kaupp, U. B. and Gensch, T. (2004). Chloride accumulation in mammalian olfactory sensory neurons. *J. Neurosci.* 24, 7931-7938. 10.1523/JNEUROSCI.2115-04.2004 15356206 PMC6729923

[JCS264325C64] Khan, S. S., Jaimon, E., Lin, Y.-E., Nikoloff, J., Tonelli, F., Alessi, D. R. and Pfeffer, S. R. (2024). Loss of primary cilia and dopaminergic neuroprotection in pathogenic LRRK2-driven and idiopathic Parkinson's disease. *Proc. Natl. Acad. Sci. USA* 121, e2402206121. 10.1073/pnas.2402206121 39088390 PMC11317616

[JCS264325C151] Kierzek, M., Deal, P. E., Miller, E. W., Mukherjee, S., Wachten, D., Baumann, A., Kaupp, U. B., Strünker, T. and Brenker, C. (2021). Simultaneous recording of multiple cellular signaling events by frequency- and spectrally-tuned multiplexing of fluorescent probes. *eLife* 10, e63129. 10.7554/eLife.63129 34859780 PMC8700268

[JCS264325C65] Kiesel, P., Viar, G. A., Tsoy, N., Maraspini, R., Gorilak, P., Varga, V., Honigmann, A. and Pigino, G. (2020). The molecular structure of mammalian primary cilia revealed by cryo-electron tomography. *Nat. Struct. Mol. Biol.* 27, 1115-1124. 10.1038/s41594-020-0507-4 32989303 PMC7610599

[JCS264325C66] Kinnebrew, M., Iverson, E. J., Patel, B. B., Pusapati, G. V., Kong, J. H., Johnson, K. A., Luchetti, G., Eckert, K. M., McDonald, J. G., Covey, D. F. et al. (2019). Cholesterol accessibility at the ciliary membrane controls hedgehog signaling. *eLife* 8, e50051. 10.7554/eLife.50051 31657721 PMC6850779

[JCS264325C67] Kinnebrew, M., Luchetti, G., Sircar, R., Frigui, S., Viti, L. V., Naito, T., Beckert, F., Saheki, Y., Siebold, C., Radhakrishnan, A. et al. (2021). Patched 1 reduces the accessibility of cholesterol in the outer leaflet of membranes. *eLife* 10, e70504. 10.7554/eLife.70504 34698632 PMC8654371

[JCS264325C68] Kirschner, M. and Gerhart, J. (1998). Evolvability. *Proc. Natl. Acad. Sci. USA* 95, 8420-8427. 10.1073/pnas.95.15.8420 9671692 PMC33871

[JCS264325C69] Kitano, H. (2004). Biological robustness. *Nat. Rev. Genet.* 5, 826-837. 10.1038/nrg1471 15520792

[JCS264325C70] Kleene, S. and Gesteland, R. (1991). Calcium-activated chloride conductance in frog olfactory cilia. *J. Neurosci.* 11, 3624-3629. 10.1523/JNEUROSCI.11-11-03624.1991 1941099 PMC6575529

[JCS264325C71] Koemeter-Cox, A. I., Sherwood, T. W., Green, J. A., Steiner, R. A., Berbari, N. F., Yoder, B. K., Kauffman, A. S., Monsma, P. C., Brown, A., Askwith, C. C. et al. (2014). Primary cilia enhance kisspeptin receptor signaling on gonadotropin-releasing hormone neurons. *Proc. Natl. Acad. Sci. USA* 111, 10335-10340. 10.1073/pnas.1403286111 24982149 PMC4104922

[JCS264325C72] Körschen, H. G., Hamzeh, H., Pascal, R., Alvarez, L., Bönigk, W., Kaur, N., Levin, L. R., Buck, J., Kambach, C., Michino, M. et al. (2021). External fertilization is orchestrated by a pH-regulated soluble adenylyl cyclase controlling sperm motility and chemotaxis. *bioRxiv*, 2021.06.18.448929. 10.1101/2021.06.18.448929

[JCS264325C73] Kostyanovskaya, E., Lasser, M. C., Wang, B., Schmidt, J., Bader, E., Buteo, C., Arbelaez, J., Sindledecker, A. R., McCluskey, K. E., Castillo, O. et al. (2025). Convergence of autism proteins at the cilium. *bioRxiv*, 2024.12.05.626924. 10.1101/2024.12.05.626924

[JCS264325C74] Kultgen, P. L., Byrd, S. K., Ostrowski, L. E. and Milgram, S. L. (2002). Characterization of an A-kinase anchoring protein in human ciliary axonemes. *Mol. Biol. Cell* 13, 4156-4166. 10.1091/mbc.e02-07-0391 12475942 PMC138623

[JCS264325C75] Kurahashi, T. and Yau, K.-W. (1993). Co-existence of cationic and chloride components in odorant-induced current of vertebrate olfactory receptor cells. *Nature* 363, 71-74. 10.1038/363071a0 7683113

[JCS264325C76] Lacey, S. E. and Pigino, G. (2025). The intraflagellar transport cycle. *Nat. Rev. Mol. Cell Biol.* 26, 175-192. 10.1038/s41580-024-00797-x 39537792

[JCS264325C77] Ladbury, J. E. and Arold, S. T. (2012). Noise in cellular signaling pathways: causes and effects. *Trends Biochem. Sci.* 37, 173-178. 10.1016/j.tibs.2012.01.001 22341496 PMC3348409

[JCS264325C78] Lange, S. M., Bennett, J. A., Eisert, R. J. and Brown, A. (2025). A conserved mechanism for the retrieval of polyubiquitinated proteins from cilia. *Cell*, S0092-8674(25)00865-7. 10.1016/j.cell.2025.07.043 PMC12380154 40840444

[JCS264325C79] Lemmon, M. A., Freed, D. M., Schlessinger, J. and Kiyatkin, A. (2016). The dark side of cell signaling: positive roles for negative regulators. *Cell* 164, 1172-1184. 10.1016/j.cell.2016.02.047 26967284 PMC4830124

[JCS264325C80] Levine, J., Kueh, H. Y. and Mirny, L. (2007). Intrinsic fluctuations, robustness, and tunability in signaling cycles. *Biophys. J.* 92, 4473-4481. 10.1529/biophysj.106.088856 17400695 PMC1877790

[JCS264325C81] Liberto, V. D., Mudò, G. and Belluardo, N. (2019). Crosstalk between receptor tyrosine kinases (RTKs) and G protein-coupled receptors (GPCR) in the brain: Focus on heteroreceptor complexes and related functional neurotrophic effects. *Neuropharmacology* 152, 67-77. 10.1016/j.neuropharm.2018.11.018 30445101

[JCS264325C82] Logothetis, D. E., Kurachi, Y., Galper, J., Neer, E. J. and Clapham, D. E. (1987). The βγ subunits of GTP-binding proteins activate the muscarinic K+ channel in heart. *Nature* 325, 321-326. 10.1038/325321a0 2433589

[JCS264325C83] Loktev, A. V. and Jackson, P. K. (2013). Neuropeptide Y family receptors traffic via the Bardet-Biedl syndrome pathway to signal in neuronal primary cilia. *Cell Rep.* 5, 1316-1329. 10.1016/j.celrep.2013.11.011 24316073

[JCS264325C84] Mangan, S. and Alon, U. (2003). Structure and function of the feed-forward loop network motif. *Proc. Natl. Acad. Sci. USA* 100, 11980-11985. 10.1073/pnas.2133841100 14530388 PMC218699

[JCS264325C85] Marley, A., Choy, R. W.-Y. and von Zastrow, M. (2013). GPR88 reveals a discrete function of primary cilia as selective insulators of GPCR cross-talk. *PLoS ONE* 8, e70857. 10.1371/journal.pone.0070857 23936473 PMC3732291

[JCS264325C86] Mayr, E. (1961). Cause and effect in biology. *Science* 134, 1501-1506. 10.1126/science.134.3489.1501 14471768

[JCS264325C87] Menco, B. and Ph, M. (1997). Ultrastructural aspects of olfactory signaling. *Chem. Senses* 22, 295-311. 10.1093/chemse/22.3.295 9218142

[JCS264325C88] Menco, B. P. and Farbman, A. I. (1987). Genesis of cilia and microvilli of rat nasal epithelia during prenatal development. III. Respiratory epithelium surface, including a comparison with the surface of the olfactory epithelium - PMC. *J. Anat.* 152, 145-160. 3654366 PMC1261753

[JCS264325C89] Mill, P., Christensen, S. T. and Pedersen, L. B. (2023). Primary cilia as dynamic and diverse signalling hubs in development and disease. *Nat. Rev. Genet.* 24, 421-441. 10.1038/s41576-023-00587-9 37072495 PMC7615029

[JCS264325C90] Moore, B. S., Stepanchick, A. N., Tewson, P. H., Hartle, C. M., Zhang, J., Quinn, A. M., Hughes, T. E. and Mirshahi, T. (2016). Cilia have high cAMP levels that are inhibited by Sonic Hedgehog-regulated calcium dynamics. *Proc. Natl. Acad. Sci. USA* 113, 13069-13074. 10.1073/pnas.1602393113 27799542 PMC5135322

[JCS264325C91] Morales, B., Barrera, N., Uribe, P., Mora, C. and Villalón, M. (2000). Functional cross talk after activation of P2 and P1 receptors in oviductal ciliated cells. *Am. J. Physiol. Cell Physiol.* 279, C658-C669. 10.1152/ajpcell.2000.279.3.C658 10942716

[JCS264325C92] Mukhopadhyay, S., Wen, X., Chih, B., Nelson, C. D., Lane, W. S., Scales, S. J. and Jackson, P. K. (2010). TULP3 bridges the IFT-A complex and membrane phosphoinositides to promote trafficking of G protein-coupled receptors into primary cilia. *Gene Dev.* 24, 2180-2193. 10.1101/gad.1966210 20889716 PMC2947770

[JCS264325C93] Mukhopadhyay, S., Wen, X., Ratti, N., Loktev, A., Rangell, L., Scales, S. J. and Jackson, P. K. (2013). The Ciliary G-protein-coupled receptor Gpr161 negatively regulates the sonic hedgehog pathway via cAMP signaling. *Cell* 152, 210-223. 10.1016/j.cell.2012.12.026 23332756

[JCS264325C94] Müller, A., Klena, N., Pang, S., Garcia, L. E. G., Topcheva, O., Duran, S. A., Sulaymankhil, D., Seliskar, M., Mziaut, H., Schöniger, E. et al. (2024). Structure, interaction and nervous connectivity of beta cell primary cilia. *Nat. Commun.* 15, 9168. 10.1038/s41467-024-53348-5 39448638 PMC11502866

[JCS264325C95] Murcia, N. S., Richards, W. G., Yoder, B. K., Mucenski, M. L., Dunlap, J. R. and Woychik, R. P. (2000). The Oak Ridge Polycystic Kidney (orpk) disease gene is required for left-right axis determination. *Development* 127, 2347-2355. 10.1242/dev.127.11.2347 10804177

[JCS264325C96] Mykytyn, K. and Askwith, C. (2017). G-protein-coupled receptor signaling in Cilia. *Cold Spring Harb. Perspect. Biol.* 9, a028183. 10.1101/cshperspect.a028183 28159877 PMC5585845

[JCS264325C97] Nechipurenko, I. V. (2020). The enigmatic role of lipids in Cilia signaling. *Front. Cell Dev. Biol.* 8, 777. 10.3389/fcell.2020.00777 32850869 PMC7431879

[JCS264325C98] Nilsson, C. I., Dumral, Ö., Sanchez, G., Xie, B., Müller, A., Solimena, M., Ren, H. and Idevall-Hagren, O. (2025). Somatostatin triggers local cAMP and Ca2+ signaling in primary cilia to modulate pancreatic β-cell function. *EMBO J.* 44, 1663-1691. 10.1038/s44318-025-00383-7 39939781 PMC11914567

[JCS264325C99] Nonaka, S., Tanaka, Y., Okada, Y., Takeda, S., Harada, A., Kanai, Y., Kido, M. and Hirokawa, N. (1998). Randomization of left–right asymmetry due to loss of nodal cilia generating leftward flow of extraembryonic fluid in mice lacking KIF3B motor protein. *Cell* 95, 829-837. 10.1016/S0092-8674(00)81705-5 9865700

[JCS264325C100] Ott, C. M. and Mukhopadhyay, S. (2025). Taking down the primary cilium: pathways for disassembly in differentiating cells. *BioEssays*. e70060. 10.1002/bies.70060 40888126 PMC12516293

[JCS264325C101] Ott, C. M., Constable, S., Nguyen, T. M., White, K., Lee, W.-C. A., Lippincott-Schwartz, J. and Mukhopadhyay, S. (2024a). Permanent deconstruction of intracellular primary cilia in differentiating granule cell neurons. *J. Cell Biol.* 223, e202404038. 10.1083/jcb.202404038 39137043 PMC11320830

[JCS264325C102] Ott, C. M., Torres, R., Kuan, T.-S., Kuan, A., Buchanan, J., Elabbady, L., Seshamani, S., Bodor, A. L., Collman, F., Bock, D. D. et al. (2024b). Ultrastructural differences impact cilia shape and external exposure across cell classes in the visual cortex. *Curr. Biol.* 34, 2418-2433. 10.1016/j.cub.2024.04.043 38749425 PMC11217952

[JCS264325C103] Ou, Y., Ruan, Y., Cheng, M., Moser, J. J., Rattner, J. B. and Hoorn, F. A. and van der, (2009). Adenylate cyclase regulates elongation of mammalian primary cilia. *Exp. Cell Res.* 315, 2802-2817. 10.1016/j.yexcr.2009.06.028 19576885 PMC3161028

[JCS264325C104] Pablo, J. L., DeCaen, P. G. and Clapham, D. E. (2017). Progress in ciliary ion channel physiology. *J. Gen. Physiol.* 149, 37-47. 10.1085/jgp.201611696 27999145 PMC5217089

[JCS264325C105] Pal, K., Hwang, S., Somatilaka, B., Badgandi, H., Jackson, P. K., DeFea, K. and Mukhopadhyay, S. (2016). Smoothened determines β-arrestin–mediated removal of the G protein–coupled receptor Gpr161 from the primary cilium. *J. Cell Biol.* 212, 861-875. 10.1083/jcb.201506132 27002170 PMC4810300

[JCS264325C106] Paolocci, E. and Zaccolo, M. (2023). Compartmentalised cAMP signalling in the primary cilium. *Front. Physiol.* 14, 1187134. 10.3389/fphys.2023.1187134 37256063 PMC10226274

[JCS264325C107] Park, K. and Leroux, M. R. (2022). Composition, organization and mechanisms of the transition zone, a gate for the cilium. *EMBO Rep.* 23, e55420. 10.15252/embr.202255420 36408840 PMC9724682

[JCS264325C108] Pearring, J. N., Salinas, R. Y., Baker, S. A. and Arshavsky, V. Y. (2013). Protein sorting, targeting and trafficking in photoreceptor cells. *Prog. Retin. Eye Res.* 36, 24-51. 10.1016/j.preteyeres.2013.03.002 23562855 PMC3759535

[JCS264325C109] Petrov, K., Magalhaes, T. A. and Salic, A. (2021). Mechanism and ultrasensitivity in Hedgehog signaling revealed by Patched1 disease mutations. *Proc. Natl. Acad. Sci. USA* 118, e2006800118. 10.1073/pnas.2006800118 33526656 PMC8017988

[JCS264325C110] Pichlo, M., Bungert-Plümke, S., Weyand, I., Seifert, R., Bönigk, W., Strünker, T., Kashikar, N. D., Goodwin, N., Müller, A., Körschen, H. G. et al. (2014). High density and ligand affinity confer ultrasensitive signal detection by a guanylyl cyclase chemoreceptor. *J. Cell Biol.* 206, 541-557. 10.1083/jcb.201402027 25135936 PMC4137060

[JCS264325C111] Pigino, G. (2021). Intraflagellar transport. *Curr. Biol.* 31, R530-R536. 10.1016/j.cub.2021.03.081 34033785

[JCS264325C112] Puck, T. T., Marcus, P. I. and Cieciura, S. J. (1956). Clonal growth of mammalian cells in vitro. *J. Exp. Med.* 103, 273-284. 10.1084/jem.103.2.273 13286432 PMC2136583

[JCS264325C113] Pugh, E. N. and Lamb, T. D. (1993). Amplification and kinetics of the activation steps in phototransduction. *Biochim. Biophys. Acta* 1141, 111-149. 10.1016/0005-2728(93)90038-H 8382952

[JCS264325C114] Ran, J., Yang, Y., Li, D., Liu, M. and Zhou, J. (2015). Deacetylation of α-tubulin and cortactin is required for HDAC6 to trigger ciliary disassembly. *Sci. Rep.* 5, 12917. 10.1038/srep12917 26246421 PMC4526867

[JCS264325C115] Reisert, J., Bauer, P. J., Yau, K.-W. and Frings, S. (2003). The Ca-activated Cl channel and its control in rat olfactory receptor neurons. *J. Gen. Physiol.* 122, 349-364. 10.1085/jgp.200308888 12939394 PMC2234486

[JCS264325C116] Reiter, J. F. and Leroux, M. R. (2017). Genes and molecular pathways underpinning ciliopathies. *Nat. Rev. Mol. Cell Biol.* 18, 533-547. 10.1038/nrm.2017.60 28698599 PMC5851292

[JCS264325C117] Rieke, F. and Baylor, D. A. (1998). Single-photon detection by rod cells of the retina. *Rev. Mod. Phys.* 70, 1027-1036. 10.1103/RevModPhys.70.1027

[JCS264325C118] Rozenfeld, R. and Devi, L. A. (2010). Exploring a role for heteromerization in GPCR signalling specificity. *Biochem. J.* 433, 11-18. 10.1042/BJ20100458 PMC3115900 21158738

[JCS264325C119] Satir, P., Mitchell, D. R. and Jékely, G. (2008). Chapter 3 how did the cilium evolve? *Curr. Top. Dev. Biol.* 85, 63-82. 10.1016/S0070-2153(08)00803-X 19147002

[JCS264325C120] Shah, A. S., Ben-Shahar, Y., Moninger, T. O., Kline, J. N. and Welsh, M. J. (2009). Motile cilia of human airway epithelia are chemosensory. *Science* 325, 1131-1134. 10.1126/science.1173869 19628819 PMC2894709

[JCS264325C121] Shi, X., Garcia, G., Weghe, J. C. V. D., McGorty, R., Pazour, G. J., Doherty, D., Huang, B. and Reiter, J. F. (2017). Super-resolution microscopy reveals that disruption of ciliary transition-zone architecture causes Joubert syndrome. *Nat. Cell Biol.* 19, 1178-1188. 10.1038/ncb3599 28846093 PMC5695680

[JCS264325C122] Shibata, T. and Fujimoto, K. (2005). Noisy signal amplification in ultrasensitive signal transduction. *Proc. Natl. Acad. Sci. USA* 102, 331-336. 10.1073/pnas.0403350102 15625116 PMC544281

[JCS264325C123] Shinde, S. R., Nager, A. R. and Nachury, M. V. (2020). Ubiquitin chains earmark GPCRs for BBSome-mediated removal from cilia. *J. Cell Biol.* 219, e202003020. 10.1083/jcb.202003020 33185668 PMC7716378

[JCS264325C124] Skiba, N. P., Lewis, T. R., Spencer, W. J., Castillo, C. M., Shevchenko, A. and Arshavsky, V. Y. (2023). Absolute quantification of photoreceptor outer segment proteins. *J. Proteome Res.* 22, 2703-2713. 10.1021/acs.jproteome.3c00267 37493966 PMC10513726

[JCS264325C125] Spivey, H. O. and Ovádi, J. (1999). Substrate channeling. *Methods* 19, 306-321. 10.1006/meth.1999.0858 10527733

[JCS264325C126] Sung, C.-H. and Leroux, M. R. (2013). The roles of evolutionarily conserved functional modules in cilia-related trafficking. *Nat. Cell Biol.* 15, 1387-1397. 10.1038/ncb2888 24296415 PMC4016715

[JCS264325C127] Sweetlove, L. J. and Fernie, A. R. (2018). The role of dynamic enzyme assemblies and substrate channelling in metabolic regulation. *Nat. Commun.* 9, 2136. 10.1038/s41467-018-04543-8 29849027 PMC5976638

[JCS264325C128] Takeuchi, H. and Kurahashi, T. (2018). Second messenger molecules have a limited spread in olfactory cilia. *J. Gen. Physiol.* 150, 1647-1659. 10.1085/jgp.201812126 30352795 PMC6279364

[JCS264325C129] Tang, T., Tan, Q., Han, S., Diemar, A., Löbner, K., Wang, H., Schüß, C., Behr, V., Mörl, K., Wang, M. et al. (2022). Receptor-specific recognition of NPY peptides revealed by structures of NPY receptors. *Sci. Adv.* 8, eabm1232. 10.1126/sciadv.abm1232 35507650 PMC9067930

[JCS264325C130] Trötschel, C., Hamzeh, H., Alvarez, L., Pascal, R., Lavryk, F., Bönigk, W., Körschen, H. G., Müller, A., Poetsch, A., Rennhack, A. et al. (2020). Absolute proteomic quantification reveals design principles of sperm flagellar chemosensation. *EMBO J.* 39, EMBJ2019102723. 10.15252/embj.2019102723 PMC7024835 31880004

[JCS264325C131] Truong, M. E., Bilekova, S., Choksi, S. P., Li, W., Bugaj, L. J., Xu, K. and Reiter, J. F. (2021). Vertebrate cells differentially interpret ciliary and extraciliary cAMP. *Cell* 184, 2911-2926.e18. 10.1016/j.cell.2021.04.002 33932338 PMC8450001

[JCS264325C132] Tu, H.-Q., Li, S., Xu, Y.-L., Zhang, Y.-C., Li, P.-Y., Liang, L.-Y., Song, G.-P., Jian, X.-X., Wu, M., Song, Z.-Q. et al. (2023). Rhythmic cilia changes support SCN neuron coherence in circadian clock. *Science* 380, 972-979. 10.1126/science.abm1962 37262147

[JCS264325C133] Tullman-Ercek, D. (2015). “Channeling” Hans Krebs. *Nat. Chem. Biol.* 11, 180-181. 10.1038/nchembio.1758 25689335

[JCS264325C134] Tulsian, N. K., Krishnamurthy, S. and Anand, G. S. (2017). Channeling of cAMP in PDE-PKA complexes promotes signal adaptation. *Biophys. J.* 112, 2552-2566. 10.1016/j.bpj.2017.04.045 28636912 PMC5479052

[JCS264325C135] Vert, G. and Chory, J. (2011). Crosstalk in cellular signaling: background noise or the real thing? *Dev. Cell* 21, 985-991. 10.1016/j.devcel.2011.11.006 22172668 PMC3281494

[JCS264325C136] Vieira, O. V., Gaus, K., Verkade, P., Fullekrug, J., Vaz, W. L. C. and Simons, K. (2006). FAPP2, cilium formation, and compartmentalization of the apical membrane in polarized Madin-Darby canine kidney (MDCK) cells. *Proc. Natl. Acad. Sci. USA* 103, 18556-18561. 10.1073/pnas.0608291103 17116893 PMC1693701

[JCS264325C137] von Zastrow, M. (2003). *Handbook of Cell Signaling*, pp. 181-186, Elsevier Science, USA.

[JCS264325C138] Vuolo, L., Herrera, A., Torroba, B., Menendez, A. and Pons, S. (2015). Ciliary adenylyl cyclases control the Hedgehog pathway. *J. Cell Sci.* 128, 2928-2937. 10.1242/jcs.172635 26092933

[JCS264325C139] Wang, C. E. and Ogden, S. K. (2025). G protein–coupled receptor signal intersection at the primary cilium. *BioEssays*. 47, e70015. 10.1002/bies.70015 40277275 PMC13244655

[JCS264325C140] Wang, H., Li, Y., Li, X., Sun, Z., Yu, F., Pashang, A., Kulasiri, D., Li, H. W., Chen, H., Hou, H. et al. (2025). The primary cilia are associated with the axon initial segment in neurons. *Adv. Sci.* 12, e2407405. 10.1002/advs.202407405 PMC11884599 39804991

[JCS264325C141] Windler, F., Bönigk, W., Körschen, H. G., Grahn, E., Strünker, T., Seifert, R. and Kaupp, U. B. (2018). The solute carrier SLC9C1 is a Na+/H+-exchanger gated by an S4-type voltage-sensor and cyclic-nucleotide binding. *Nat. Commun.* 9, 2809. 10.1038/s41467-018-05253-x 30022052 PMC6052114

[JCS264325C142] Wong, S. T., Trinh, K., Hacker, B., Chan, G. C., Lowe, G., Gaggar, A., Xia, Z., Gold, G. H. and Storm, D. R. (2000). Disruption of the type III adenylyl cyclase gene leads to peripheral and behavioral anosmia in transgenic mice. *Neuron* 27, 487-497. 10.1016/S0896-6273(00)00060-X 11055432

[JCS264325C143] Xiong, W. and Ferrell, J. E. (2003). A positive-feedback-based bistable ‘memory module’ that governs a cell fate decision. *Nature* 426, 460-465. 10.1038/nature02089 14647386

[JCS264325C144] Xun, Y., Jiang, Y., Xu, B., Tang, M., Ludwig, S., Nakamura, K., Mukhopadhyay, S., Liu, C., Beutler, B. and Zhang, Z. (2025). GPR45 modulates Gαs at primary cilia of the paraventricular hypothalamus to control food intake. *Science* 388, eadp3989. 10.1126/science.adp3989 40472089 PMC12704111

[JCS264325C145] Yang, T. T., Su, J., Wang, W.-J., Craige, B., Witman, G. B., Tsou, M.-F. B. and Liao, J.-C. (2015). Superresolution pattern recognition reveals the architectural map of the ciliary transition zone. *Sci. Rep.* 5, 14096. 10.1038/srep14096 26365165 PMC4568515

[JCS264325C146] Yang, S., Bahl, K., Chou, H.-T., Woodsmith, J., Stelzl, U., Walz, T. and Nachury, M. V. (2020). Near-atomic structures of the BBSome reveal the basis for BBSome activation and binding to GPCR cargoes. *eLife* 9, e55954. 10.7554/eLife.55954 32510327 PMC7311171

[JCS264325C147] Yau, K.-W. and Hardie, R. C. (2009). Phototransduction motifs and variations. *Cell* 139, 246-264. 10.1016/j.cell.2009.09.029 19837030 PMC2885920

[JCS264325C148] Ye, F., Nager, A. R. and Nachury, M. V. (2018). BBSome trains remove activated GPCRs from cilia by enabling passage through the transition zone. *J. Cell Biol.* 217, 1847-1868. 10.1083/jcb.201709041 29483145 PMC5940304

[JCS264325C149] Zhao, H., Khan, Z. and Westlake, C. J. (2023). Ciliogenesis membrane dynamics and organization. *Semin. Cell Dev. Biol.* 133, 20-31. 10.1016/j.semcdb.2022.03.021 35351373 PMC9510604

[JCS264325C150] Zhu, B. T. (1993). The competitive and noncompetitive antagonism of receptor-mediated drug actions in the presence of spare receptors. *J. Pharmacol. Toxicol. Methods* 29, 85-91. 10.1016/1056-8719(93)90055-J 8318718

